# Transcriptome Analysis of *Bombyx mori* Larval Midgut during Persistent and Pathogenic Cytoplasmic Polyhedrosis Virus Infection

**DOI:** 10.1371/journal.pone.0121447

**Published:** 2015-03-27

**Authors:** Anna Kolliopoulou, Filip Van Nieuwerburgh, Dimitrios J. Stravopodis, Dieter Deforce, Luc Swevers, Guy Smagghe

**Affiliations:** 1 Insect Molecular Genetics and Biotechnology, Institute of Biosciences and Applications, National Centre for Scientific Research “Demokritos”, Aghia Paraskevi, Athens, Greece; 2 Laboratory of Pharmaceutical Biotechnology, Department of Pharmaceutics, Faculty of Pharmaceutical Sciences, Ghent University, Ghent, Belgium; 3 Department of Cell Biology and Biophysics, Faculty of Biology, University of Athens, Athens, Greece; 4 Laboratory of Agrozoology, Department of Crop Protection, Faculty of Bioscience Engineering, Ghent University, Ghent, Belgium; University of Missouri, UNITED STATES

## Abstract

Many insects can be persistently infected with viruses but do not show any obvious adverse effects with respect to physiology, development or reproduction. Here, *Bombyx mori* strain Daizo, persistently infected with cytoplasmic polyhedrosis virus (BmCPV), was used to study the host’s transcriptional response after pathogenic infection with the same virus in midgut tissue of larvae persistently and pathogenically infected as 2nd and 4th instars. Next generation sequencing revealed that from 13,769 expressed genes, 167 were upregulated and 141 downregulated in both larval instars following pathogenic infection. Several genes that could possibly be involved in *B*. *mori* immune response against BmCPV or that may be induced by the virus in order to increase infectivity were identified, whereas classification of differentially expressed transcripts (confirmed by qRT-PCR) resulted in gene categories related to physical barriers, immune responses, proteolytic / metabolic enzymes, heat-shock proteins, hormonal signaling and uncharacterized proteins. Comparison of our data with the available literature (pathogenic infection of persistently vs. non-persistently infected larvae) unveiled various similarities of response in both cases, which suggests that pre-existing persistent infection does not affect in a major way the transcriptome response against pathogenic infection. To investigate the possible host’s RNAi response against BmCPV challenge, the differential expression of RNAi-related genes and the accumulation of viral small RNAs (vsRNAs) were studied. During pathogenic infection, siRNA-like traces like the 2-fold up-regulation of the core RNAi genes *Ago-2* and *Dcr-2* as well as a peak of 20 nt small RNAs were observed. Interestingly, vsRNAs of the same size were detected at lower rates in persistently infected larvae. Collectively, our data provide an initial assessment of the relative significance of persistent infection of silkworm larvae on the host response following pathogenic infection with CPV, while they also highlight the relative importance of RNAi as an antiviral mechanism.

## Introduction

Interest in viruses originates mainly from their role as pathogens that can cause significant disease in hosts. Regarding pathogenic infections in insects, virus research focuses on the protection of beneficial insects such as the honeybee, *Apis mellifera*, and the silkworm, *Bombyx mori*, against virus infection [1, 2] as well as on the improvement of the use of viruses to control agricultural pests [3]. In addition, many research activities focus on the transmission by mosquitoes of arbovirus infections that cause disease in humans and livestock [4, 5].

With the advent of next generation sequencing technologies, however, it has been realized that many insect species can be persistently infected with viruses that do not cause an obvious adverse symptom or abnormal phenotype [6, 7]. In contrast to acute infections, which are limited in time, resulting either in clearance of the virus or death of the host, persistent infections can be maintained in the hosts for very long periods and transmitted to the offspring [8]. With time, mutualistic relationships can even be developed between host and virus and, in some cases, it has been documented that viral genes and mechanisms are adopted by the host for its own benefit, for instance in the immune response [9]. Of great interest are also arbovirus infections in mosquito vectors, which can be considered as persistent infections where both mosquito vector and virus need to survive for a sufficient time to allow transmission to the vertebrate link in the infection cycle [10]. Also honeybee populations can be persistently infected with multiple viruses without affecting the health of the colony [11]. However, bee viruses are often mentioned as one of the multifactorial causes of the decline of honeybee colonies [12] and it can therefore be hypothesized that persistent infections could turn pathogenic in conditions of increased stress. Because of their prevalence, persistent infections deserve more attention with respect to alterations in the physiology of the host, such as the immune response, and to conditions that could turn apparently harmless persistent infections into disease.

*B*. *mori* cytoplasmic polyhedrosis virus (BmCPV), a reovirus characterized by a segmented dsRNA genome, is a major pathogen of the silkworm [13]. In contrast to baculovirus infections, which can spread efficiently throughout the body and are very virulent to the host, CPV infections are mostly or exclusively limited to the midgut tissue, cause less damage and therefore have a propensity to become persistent [14]. During our research with BmCPV, it was noticed that our laboratory strain of silkworm, Daizo, was persistently infected with BmCPV. Despite the infection, however, animals appeared healthy, showed normal growth and metamorphosis, and produced batches of fertilized eggs of typical size. Because of this observation, we considered the persistently infected silkworm strain as an interesting experimental model to investigate the (alteration of the) transcriptional response after feeding of a high dose of BmCPV polyhedra that caused clear pathogenic effects. Several studies concerning the transcriptional responses to BmCPV infection can be found in literature; however, these responses were recorded in larvae for which no persistent infection was reported [15–18]. Another study was focused on the miRNAs’ differential expression during BmCPV infection [19].

In our study, deep sequencing (Illumina) technology was applied to obtain an initial assessment of the transcriptional response in silkworm larvae that were persistently or pathogenically infected with BmCPV. Instead of having different biological replicates at a single developmental stage, it was decided in this explorative study to compare the transcriptional responses between persistently and pathogenically infected animals at two different developmental stages (2^nd^ and 4^th^ larval instars) and to focus on genes that become differentially expressed irrespective of the developmental stage. The identification of differentially expressed genes was subsequently validated by qRT-PCR experiments on samples obtained from 2^nd^ instar larvae. Despite the limitation of the deep sequencing analysis (data from four unique samples, corresponding to persistent or pathogenic infection in the 2^nd^ or 4^th^ instar), it is believed that valuable preliminary data were obtained that will stimulate further research. It is noted that a similar format of experimental design was applied to the study of differentially expressed microRNAs (miRNAs) after BmCPV infection [19].

Our study establishes a considerable overlap in the transcriptional response to BmCPV during a pathogenic infection of persistently infected silkworm larvae with the transcriptional response documented in the above mentioned studies where no persistent infection was reported. In addition, we focused on the involvement of the RNA interference (RNAi) machinery during BmCPV infection, which has not received much attention in previous BmCPV-related studies. Although RNAi has been considered as the most important antiviral response in *Drosophila* and mosquitoes [20–22], in insects of other groups, such as the honeybee or the silkworm, its involvement in antiviral defense remains largely unknown. Attention is therefore paid to the transcriptional response of RNAi machinery genes during a pathogenic BmCPV infection as well as to the detection of virus-derived small RNAs (vsRNAs).

## Materials and Methods

### Silkworm rearing and infection with BmCPV

The larvae of *B*. *mori*, Daizo strain, were reared on artificial diet (Yakuruto, Tokyo, Japan) at 25°C under a photoperiod of 12 h light and 12 h dark. For pathogenic infection with BmCPV, aliquots of artificial diet were treated with 50 μl of a concentrated solution of polyhedra of BmCPV (3.25 x 10^7^ polyhedra/ml) and fed to 2^nd^ instar (N = 30) or 4^th^ instar larvae (N = 30), 1–2 days after molt. Successful pathogenic infection was indicated by retardation of growth and confirmed by detection of polyhedra by means of optical microscopy as well as by gene-specific RT-PCR for *polyhedrin* (see Results and Discussion section). Midgut and body wall tissue was collected from pathogenically infected larvae at 10–20 days after feeding of BmCPV polyhedra. The same tissues were also collected from control (persistently infected) larvae at a similar stage.

### RNA extraction and reverse-transcription PCR (RT-PCR)

Tissues were homogenized in TRI Reagent (Sigma, Saint Louis, MO) and total RNA was extracted according to the manufacturer's protocol. The quantity of extracted RNA was assessed with a NanoDrop 1000 Spectrophotometer (Thermo Scientific, Waltham, MA) and/or by electrophoresis on 1% (w/v) agarose gels. RNA for specific detection of BmCPV *polyhedrin* was first mixed with DMSO at 1:1 ratio and heated at 50°C for 45 minutes for denaturation of dsRNA BmCPV genome. One microgram of total RNA was used as template for first-strand complementary DNA (cDNA) synthesis as performed by a Revert Aid reverse transcriptase (Thermo Scientific). Random hexamers (Thermo Scientific) were utilized as primers for cDNA templates of quantitative RT-PCR, whereas BmCPV segment 10-specific primer 5’-AGGATCATGGCAGACGTAGC-3’ was used to synthesize cDNA templates for *polyhedrin*-specific RT-PCR. Viral *polyhedrin* was detected using the same RT primer as forward primer and 5’-TAGGCGTTCGGCGAAATGT-3’ as reverse primer, under the following cycling conditions: 94°C for 30 s, 50°C for 30 s and 72°C for 45 s (40 cycles).

### Deep sequencing of RNA samples from midguts of pathogenically and persistently infected larvae

The four RNA samples that were prepared for deep sequencing analysis were derived from midgut tissue of persistently (“control”) and pathogenically infected 2^nd^ instar larvae (2c and 2inf), as well as of persistently (“control”) and pathogenically infected 4^th^ instar larvae (4c and 4inf). RNA quantifications were performed using Qubit fluorometry (Life technologies, Carlsbad, CA).

For each sample, an Illumina mRNA sequencing library was made from 100 ng of total RNA using the TruSeq Stranded mRNA Sample Prep Kit (Illumina, San Diego, CA), whereas approximately 500 ng of total RNA was used to create a small RNA library using the TruSeq Small RNA Sample Preparation Kit (Illumina). The 4 mRNA and the 4 small RNA libraries were each equimolarly pooled and sequenced in one lane of an Illumina HiSeq 2000 flowcell, generating 1 x 50 bp reads. After sequencing, the data was demultiplexed using the sample specific nucleotide barcodes. On average, 30 x 10^6^ mRNAs were generated. The mRNA and small RNA differential expression analysis was performed using CLC bio (Qiagen, Venlo, The Netherlands). All reads were trimmed for Illumina adapter sequences.

The mRNA reads were mapped to the *Bombyx* annotated genome (Kaikobase: http://sgp.dna.affrc.go.jp/pubdata/genomicsequences.html) [23]. For the 2inf, 2c, 4inf and 4c mRNA samples, the percentage of mapped reads was 24%, 37%, 29% and 36%, respectively. The lower percentages of reads that were mapped in the infected samples likely reflect the predominance of transcripts that could be mapped to the viral genome, which illustrates the severity of the infection in those samples. The number of mRNA reads that mapped to a transcript was divided by the transcript length and normalized per sample by the number of mapped reads to calculate the RPKM (Reads Per Kilobase per Million mapped reads) expression values for each gene, thus making possible the direct comparison of differential expression among samples [24, 25]. Because of the lack of biological repeats, we did not use T statistics to identify significant differentially expressed genes. Instead, we filtered the RNA-seq data according to the following criteria: (i) total gene reads count resulting from all 4 libraries should be more than 10, and (ii) selected transcripts should present at least a 2-fold RPKM change (|log2Ratio| ≥ 1) between pathogenically and persistently infected in both library pairs (2inf/2c, 4inf/4c) towards the same direction, i.e. up- or down-regulation [26, 27]. These criteria therefore ensure, first, that all relevant genes including those with low, but significant, expression level are evaluated, and, second, that a significant change in expression occurs that is independent of the developmental stage of the infected larvae.

Regarding small RNAs, after trimming, more than 95% of the original number of reads was used for small RNA tag counting. Approximately 15% of the tags had a count lower than 5 and these were discarded. The remaining tags were counted and annotated using the *Bombyx* miRNA miRBASE 19 database. The small RNAs that did not map against the *Bombyx* miRNA miRBASE 19 database were subsequently mapped against the BmCPV genome to find vsRNAs originating from the virus. Small RNA length distribution graphs were produced in R [28] by use of the viRome package (http://www.ark-genomics.org/bioinformatics/virome). The sequences from the mRNA and small RNA libraries of all 4 samples were submitted to the European Nucleotide Archive (Accession Number PRJEB7502).

### Gene Ontology (GO) analysis

A list of sequences that were identified as differentially expressed between persistently and pathogenically infected larvae (see Results and Discussion section) were analyzed for GO annotation [http://www.geneontology.org [29]]. Sequences were identified based on the annotated gene set at Kaikobase and genes resulting from the homology searches were used as input in DAVID [http://david.abcc.ncifcrf.gov [30]].

### Real-time quantitative RT-PCR

Real-time qRT-PCR was performed in a Mx3000P QPCR System (Agilent Technologies, Santa Clara, CA) equipped with MxPro QPCR Software (Agilent Technologies) using KAPA SYBR FAST qPCR Kit (Kapa Biosystems, Wilmington, MA), gene-specific primers at a final concentration of 0.55 μM each and 12.5 ng of midgut cDNA template. Relative transcript levels were normalized to the expression level of the cellular *actin* gene. PCR cycling started with initial activation of KAPA SYBR FAST Master Mix polymerase at 95°C for 3 min, followed by 40 cycles of 95°C for 5 s, 59 or 60°C for 30 s and 72°C for 5 s. Forward and reverse primers to detect specific transcripts (S1 Table) were designed using Primer Express 2.0 software (Applied Biosystems/Life Technologies). Relative expression levels of target gene (X) were calculated in relation to the transcription levels of the *actin* reference gene (R), as 2^-ΔCt^, where ΔC_t_ = C_t_^X^-C_t_^R^.

## Results and Discussion

### Persistent (non-pathogenic) infection of silkworm with BmCPV

**Fig 1:**
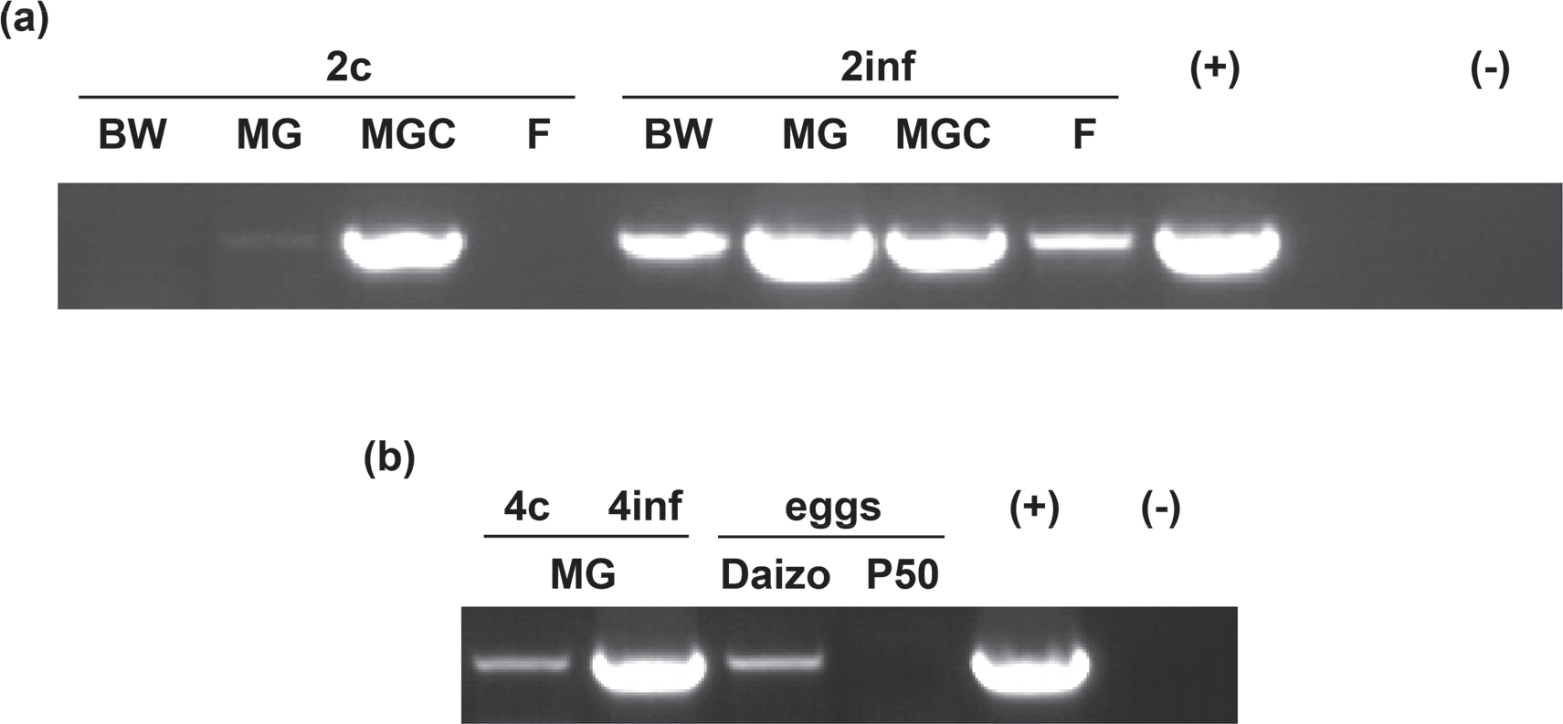
Detection of BmCPV *polyhedrin* by RT-PCR in persistently and pathogenically infected silkworm tissues and eggs. *Polyhedrin* RT-PCR was performed using samples derived from larvae persistently and pathogenically infected (a) at the 2^nd^ or (b) the 4^th^ instar (2c, 2inf, 4c and 4inf samples), as well as from Daizo and P50 silkworm strain-derived eggs. *Polyhedrin* PCR product with a size of 689 nucleotides was obtained after 40 cycles of PCR. Abbreviations: BW: body wall, MG: midgut, MGC: midgut content, F: feces, (+): positive control (BmCPV polyhedra), (-): negative control (water sample).

To verify infection of silkworm larvae with BmCPV, we performed RT-PCR with gene-specific primers for the viral *polyhedrin* gene. The samples tested corresponded to RNA collected from body wall, midgut, midgut content and feces. For pathogenic infections (2inf and 4inf samples), *polyhedrin* product was detected at high levels in all samples analyzed (midgut tissue, midgut content, feces and body wall; Fig. 1). However, rather high amounts of *polyhedrin* RNA could also be detected in the midgut content of the control (not pathogenically infected) 2^nd^ instar larvae (2c sample) and low levels were also present in the midgut tissue (both 2^nd^ and 4^th^ instar stages; Fig. 1). In addition, cDNA samples derived from the eggs of the same strain as the larvae (Daizo) were also positive for *polyhedrin* RNA, in contrast to the eggs of the P50 strain, which gave no PCR product (Fig. 1). PCR products obtained from control midguts (4^th^ instar) and Daizo eggs were validated by sequencing to correspond to BmCPV *polyhedrin*. The detection of *polyhedrin* RNA in midgut tissue and eggs of animals of the Daizo strain in our colony is indicative of a persistent state of infection by BmCPV.

**Fig 2:**
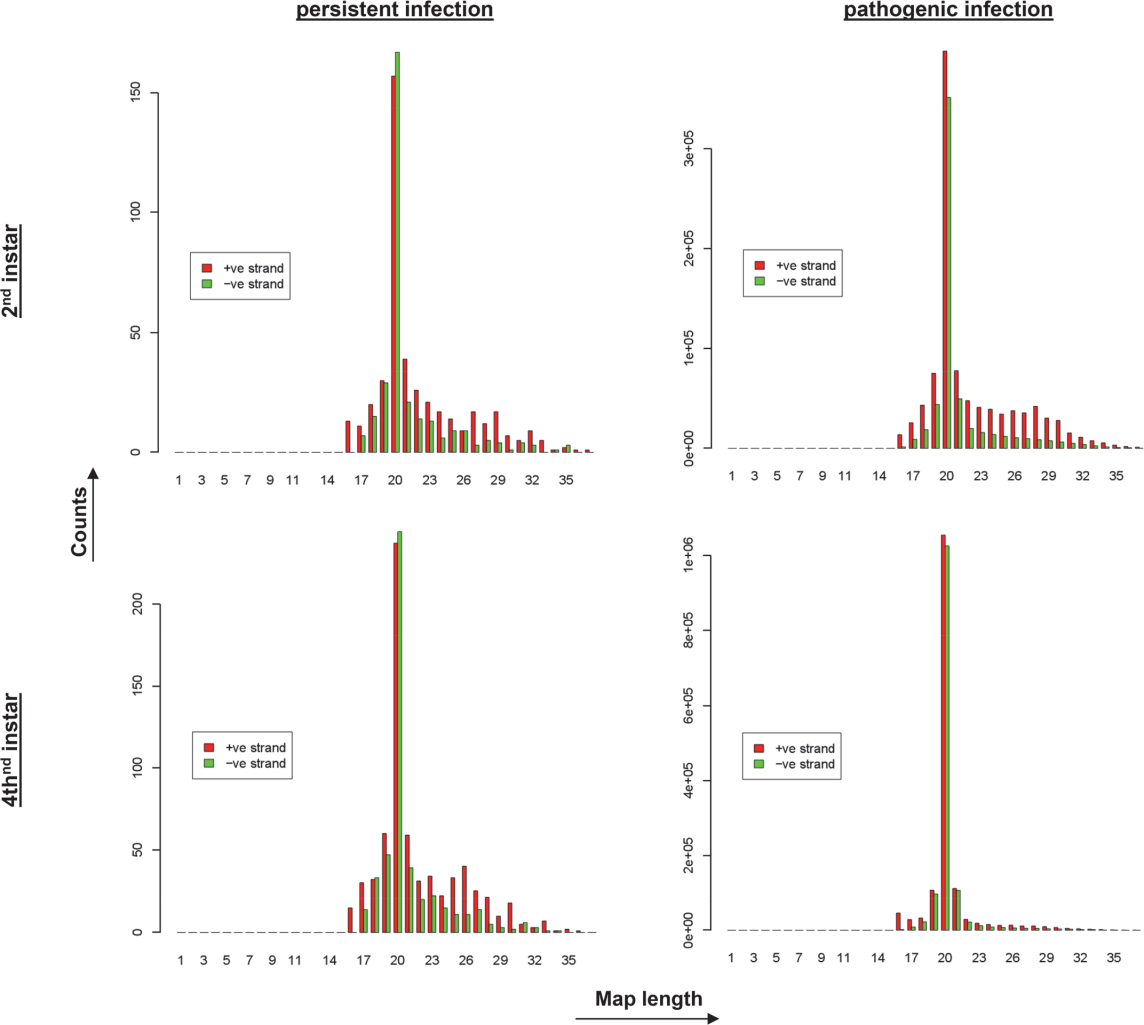
Size distribution of viral small RNA (vsRNA) reads in persistently and pathogenically BmCPV-infected midgut tissue. Graphs represent read counts that match to the positive or negative strand of BmCPV in persistently and pathogenically infected midguts of 2^nd^ and 4^th^ instar larvae. Numbers of reads (counts) are plotted against the length of the vsRNAs. A clear peak of 20 nt is observed in all samples. Please note also the differences in read counts between persistently (150–200 scale) and pathogenically infected samples (3 x 10^5^–1 x 10^6^ scale).

Thus, larvae of the Daizo strain that were pathogenically infected in our study with BmCPV were already persistently infected with the same virus at low levels. This persistent state of infection was not visibly correlated with pathogenic effects, as larvae apparently grew, molted and metamorphosed normally. It should also be mentioned that polyhedra were never detected in any control animal under normal rearing following microscope observation. Furthermore, RT-PCR experiments revealed the presence of *polyhedrin* RNA mainly in the midgut content of control larvae and only traces in the midgut tissue itself (Fig. 1), which suggests that in persistently infected larvae, the infection of the midgut tissue (epithelium and associated muscle and connective tissue) is efficiently cleared, thus preventing the occurrence of pathogenic defects. This contrasts to the situation in pathogenically infected larvae, where high amounts of *polyhedrin* RNA were detected in the midgut tissue (Fig. 1), being indicative of high levels of viral replication/production (see further below). Finally, virus-derived small RNAs (vsRNAs) were detected in both persistently and pathogenically infected larvae (Fig. 2; see also Detection and preliminary analysis *of vsRNAs* paragraph); however, the detection levels in the control larvae were minimal, further corroborating the low levels of viral RNA in persistently infected tissues.

### Oral infection of silkworm larvae with BmCPV polyhedra

Persistently infected larvae of Daizo strain were pathogenically infected through the oral route with 5.5 x 10^4^ polyhedra per larva. At the time of infection, the larvae were at the beginning of the 2^nd^ or the 4^th^ instar developmental stage. The infection was verified macroscopically by observation of the infected larvae (S1 Fig.), as well as microscopically by detection of viral polyhedra in the larval midgut tissue (S2 Fig.). As shown for a representative group of persistently and pathogenically infected larvae in S1 Fig., the sizes of the pathogenically infected larvae varied notably among several individuals. This phenomenon was observed in both 2^nd^ and 4^th^ instar infected larvae. However, polyhedra could be detected in larvae of all sizes, indicating that the impact of the infection on body size did not correlate with the presence of infection.

According to the literature, BmCPV targets epithelial midgut cells and is not expected to expand all over the larval body. Nevertheless, following observation of several larval organs under the microscope, we have detected polyhedra also in the interior of the body wall, as well as in the hemolymph (S2 Fig.).

### Transcriptome analysis of larval midgut samples

**Fig 3:**
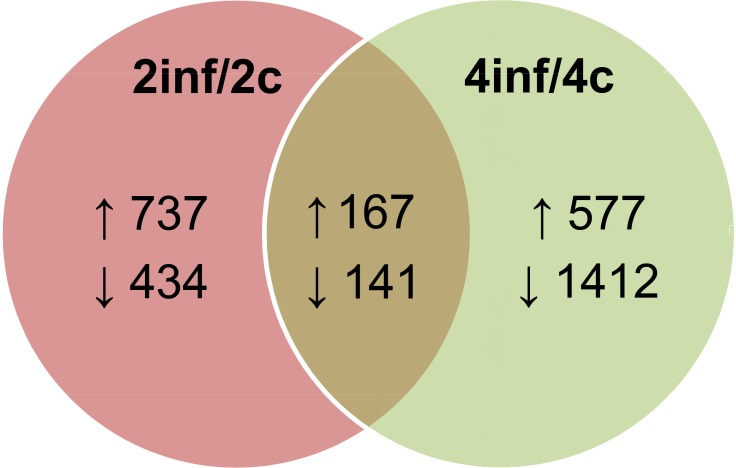
Differentially expressed genes following pathogenic infection in midgut, as determined by deep sequencing analysis. Numbers of up-regulated and down-regulated genes are indicated, after comparison of RPKMs of identified genes between 2inf and 2c libraries (2^nd^ instar), and between 4inf and 4c libraries (4^th^ instar). The numbers of genes that were up-regulated or down-regulated in both developmental stages are indicated in the section of the two diagrams.

RNA-seq was used to analyze midgut samples from silkworm larvae infected in the 2^nd^ and 4^th^ instar stage, in order to detect differentially expressed transcripts during persistent and pathogenic BmCPV infection. Deep sequencing was carried out on samples from two different developmental stages to identify transcripts that are differentially expressed irrespective of the developmental stage, instead of having biological replicates at the same stage of development. Transcriptome analysis resulted in the identification of 13,769 expressed genes over all 4 RNA samples (S1 Dataset). Filtering of RNA-seq data according to the criteria outlined in *Materials and Methods* section resulted in 167 up-regulated and 141 down-regulated transcripts in both instars (Fig. 3; S2 and S3 Datasets).

**Fig 4:**
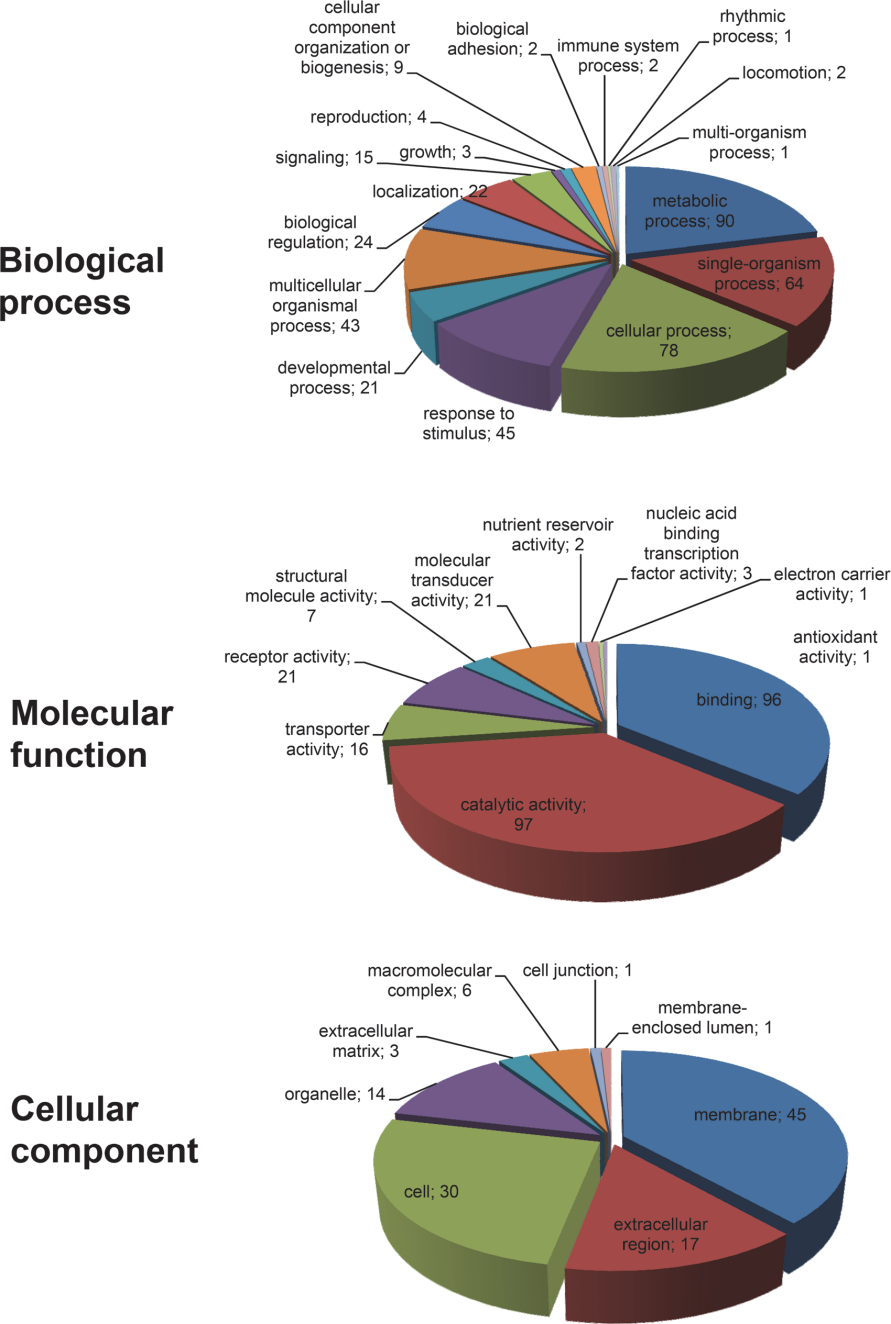
Gene ontology (GO) analysis of differentially expressed genes in pathogenically infected larvae. Genes that were differentially expressed during pathogenic infection at both 2^nd^ and 4^th^ instar developmental stages were analyzed using GO tools and categorized according to biological process, molecular function and cellular component classes. The numbers of genes that could be assigned to the different categories are indicated.

According to GO analysis of the 308 differentially expressed transcripts, 201 were found to have a GO annotation and were initially categorized in the three main GO functional groups, i.e. biological process, molecular function and cellular component. Genes belonging to each group were further classified at level 2 for each functional group (Fig. 4).

Gene transcripts found to have at least one”biological process” annotation (426), were further classified in 17 subgroups, with the most important of them belonging to metabolic process (21.1%), cellular process (18.3%), single-organism process (15%), response to stimulus (10.6%) and multicellular organismal process (10.1%) (Fig. 4; S3 Fig.). Of note is that the category “immune system process” was poorly represented. Gene transcripts with annotations of molecular function (265) were further classified in 10 categories, among which catalytic activity (36.6%) and binding (36.2%) represented the most numerous groups. It should be noted that different polymerase (including RNA-directed DNA polymerase), nucleic acid binding and nuclease activities had high rankings (Fig. 4; S3 Fig.). Finally, 117 gene sequences were annotated as cellular components and were further categorized in 8 groups. Membrane (38.5%), cell (25.6%), extracellular region (14.5%) and organelle (12%) were the four main groups (Fig. 4; S3 Fig.).

### Differentially expressed genes and their possible connection to a BmCPV-specific antiviral response

**Fig 5:**
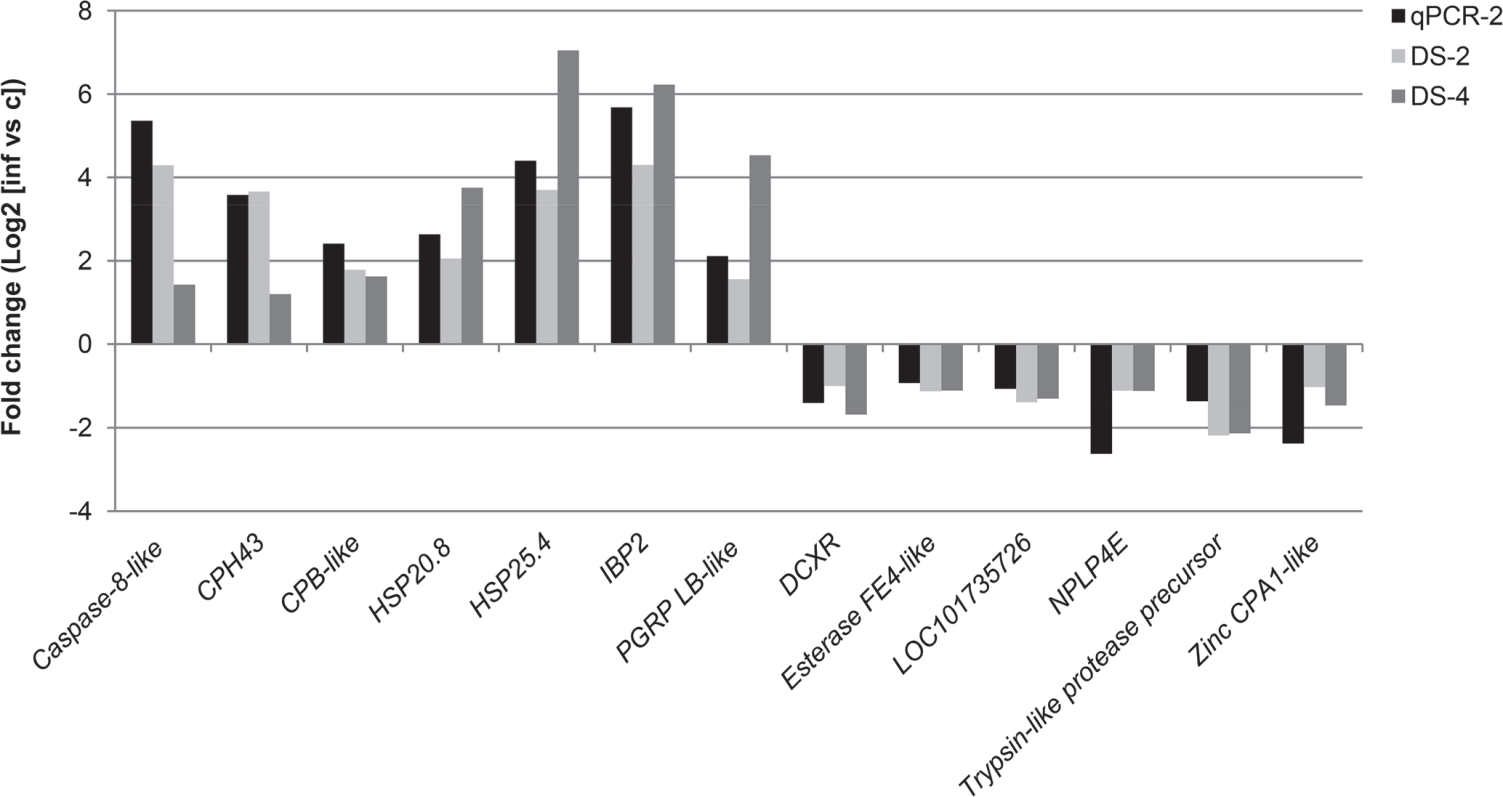
Expression levels of selected genes from pathogenically infected midguts as analyzed by deep sequencing and validated by qRT-PCR. Indicated is the fold change in expression of selected genes between persistent and pathogenic infection as obtained by qRT-PCR on 2^nd^ instar samples (qPCR-2) or by deep sequencing on both 2^nd^ and 4^th^ instar samples (DS-2 and DS-4, respectively). Selected genes belong to the group with highest difference in expression between persistent and pathogenic infection. Please note that fold changes are expressed as log2 values (a 2-fold up- or down-regulation corresponds to a log2 value of 1 or -1, respectively). See Table 2 for further explanation on gene identity and function.

Several genes possibly implicated in silkworm’s response against BmCPV infection were found by deep sequencing to be highly differentially expressed between persistently and pathogenically infected samples (S2 and S3 Datasets). Some of the most interesting genes were further validated by qRT-PCR (Fig. 5; S4 Fig.). These genes fall in several categories, i.e. physical barriers, immune responses, proteolytic enzymes, heat-shock proteins (Table 1), metabolic enzymes, hormonal signaling and uncharacterized, as outlined in detail in Table 2. Together, they constitute a complex response to BmCPV infection in the silkworm larvae.

**Table 1:** Relative RPKMs of genes coding for heat-shock proteins (HSPs).

Name	Gene ID	2inf vs 2c RPKM	4inf vs 4c RPKM
HSP19.9	BGIBMGA004540	**8.18**	**20.38**
HSP20.8	BGIBMGA004605	**4.14**	**13.48**
HSP70	BGIBMGA006313	**2.11**	**1.97**
	BGIBMGA004614	**2.37**	**2.19**
HSP70, HSC70	BGIBMGA002381	0.96	0.98
sHSP 19.1	BGIBMGA004606	0.88	**2.10**
sHSP 19.5	BGIBMGA013545	**0.58**	**8.33**
sHSP 20.2	BGIBMGA005784	**4.52**	na
sHSP 20.4	BGIBMGA004541	**2.71**	na
sHSP 21.4	BGIBMGA000944	1.02	1.02
sHSP 22.6	BGIBMGA004103	1.03	1.15
sHSP 23.8	BGIBMGA004515	**2.78**	**1.88**
sHSP 24.2	BGIBMGA005780	**3.01**	0.75
sHSP 26.6	BGIBMGA005755	**2.09**	**2.18**
sHSP 27.4	BGIBMGA005823	**1.97**	**5.26**
sHSP 42.3	BGIBMGA004101	0.99	**0.44**

Ratios of RPKM values from pathogenically versus persistently infected midgut tissue of 2^nd^ and 4^th^ instar larvae obtained by deep sequencing analysis (2c, 2inf, 4c and 4inf samples) are presented for heat-shock protein genes. Genes presenting higher than 1.5-fold up- or down-regulation are marked with bold letters. Abbreviation: na: not applicable.

**Table 2:** Selected highly differentially expressed genes following pathogenic infection of midgut tissue.

Category/ Name	Known/ Possible function
**Physical barrier**	
*CPH43*	putative cuticle gene; involved in larval molting [31]
**Immune responses**	
*IBP2*	highly homologous to IGFBP7 in vertebrates (tumor-suppressive function leading to apoptosis); involved in immune and endocrine responses [32–37]
	insulin signaling pathway is reported to be involved in the defense against pathogens in *C*. *elegans* [38]
	vertebrate insulin activates the extracellular signal-regulated kinase (ERK) in the mosquito gut; has an antiviral role in *Drosophila* cells and insect gut epithelium [39]
*PGRP LB-like*	behaves as an amidase; hydrolyzes Gram^-^ bacteria peptidoglycan and is activated by them; indirectly acts as negative regulator of the IMD pathway, thus balancing homeostasis and immune response activation [40, 41]
**Proteolytic enzymes**	
*Caspase-8-like*	death receptor-associated initiator caspase-8 is expected to be activated upon reovirus infection in the case of reovirus-induced apoptosis [42–45]
*CPB*	up-regulated in the midgut of *Anopheles gambiae* mosquitoes infected with *Plasmodium falciparum* parasite, significantly reduced growth and development of rodent parasite *P*. *berghei* in mosquitos fed on infected mice immunized against CPB [46]
*Trypsin-like protease precursor*	alkaline protease; active in the silkworm midgut alkaline environment; participates in the hydrolysis of incoming food and probably also of viral polyhedra, thus mediating release of occluded viruses and infection of midgut columnar epithelial cells [47]
	trypsin-like protease is down-regulated in BmDNV-Z and BmNPV-infected silkworm strains susceptible to the respective viruses, up-regulated in BmNPV-infected silkworm strain [47, 48]
	facilitates DENV-2 virus infection in *Aedes aegypti* [49]
*Zinc carboxypeptidase A1-like (CPA1)*	Metallocarboxypeptidase ACI, a zinc carboxypeptidase A1 (CPA1) inhibitor, is expressed by *Ascaris* (human intestinal parasite) to enhance its survival during infection [50]
**Heat-shock proteins**	
*HSPs; HSCs*	molecular chaperones in various cellular processes; danger signals thus activating host immune response [51, 52]
	HSPs and HSCs are necessary for efficient BmNPV proliferation in *Bombyx* cells as well as for PCV2 virus expansion in porcine cells [53–55]
	sHSPs are induced in BmCPV-infected larval midguts [18]
	Hsp25 has antiviral role in reovirus-infected murine cells [56]; *HSC70* is up-regulated in BmCPV-infected silkworms (72hpi) [18]; Hsc70t and HSP105 are induced by reovirus infection in murine cells [57]
**Metabolic enzymes**	
*DCXR*	converts L-xylulose in xylitol (carbohydrate metabolism); reduces the highly reactive α-dicarbonyl compounds (DCs) with endogenous/ xenobiotic origin (detoxifying enzyme) [58]
	DHS-21 (DCXR ortholog) is essential for normal life-span and reproduction of *C*. *elegans* [59]
*Esterase FE4-like*	esterases hydrolyze and inactivate insecticides (insecticide resistance), but also metabolize pathogen-secreted toxic compounds (host response) [60]; as counter-mechanism, pathogens may synthesize inhibitors against such esterase function to cause down-regulation of esterase genes
**Hormonal signaling**	
*NPLP4E*	neuropeptides: signaling molecules playing key roles in insects due to their involvement in developmental, reproductive, metabolic and behavioral processes; expressed in several tissues (brain, epidermis, ovary, prothoracic gland); possibly involved in molting regulation [61]
	*NPLP4E* maybe carries out additional functions in the midgut (here was first detected to be expressed) [31, 62]
**Uncharacterized**	
*LOC101735726*	possible role in the physiological process of DNA replication and maintenance (S5 Fig.) which may be hindered due to BmCPV presence in the midgut

The list represents genes with high differential expression levels during pathogenic infection detected by deep sequencing analysis that were also validated by qRT-PCR. The selected genes are categorized according to their function. Relevant studies representing similar responses to infection are also listed.

In the literature, several reports exist with respect to the transcriptome response to BmCPV infection using larvae without persistent virus infections [15–18]. Table 3 presents a list of all differentially expressed genes obtained from previously published studies and from our study. This list contains only genes of which the differential expression was confirmed by qRT-PCR. In accordance with all four studies, down-regulation of expression of several digestive enzymes was noted; an induction of a small set of immune response genes, of which *insulin-binding protein 2* (*ibp2*) gene was identified as significantly induced in three independent studies ([16, 18]; our study); an induction of the apoptosis pathway or repression of apoptosis inhibitors in four studies ([15–17]; our study) was shown, as well as a role for remodeling of the midgut epithelium during infection (Table 3). For the latter process, it has been speculated that the induction of the cuticle protein-like gene *CPH43*, as observed in our study, plays a role. In two studies, induction of the stress response, represented by small heat-shock protein genes (Table 1), was observed ([18]; our study). The two studies by Gao et al. [15, 16] also emphasized the possible role of the calcium-signaling pathway to induce apoptosis in infected cells, but the calcium signaling pathway genes were not among the most differentially genes that were analyzed by qRT-PCR in the other studies.

**Table 3:** Classification of genes that are induced or repressed following pathogenic BmCPV infection: comparison among different relevant studies.

Genes	SH (p50) ^[^ ^17^ ^]^	MA (p50) ^[^ ^18^ ^]^	DS (4008) ^[^ ^15^ ^]^	DS (4008) ^[^ ^16^ ^]^	DS (p50) ^[^ ^16^ ^]^	D (DaiSzo) our study
**digestive enzymes (proteases, lipases, nucleases)**						
*Acidic lipase*			←			
*Alkaline nuclease*	→					
*Chymotrypsin-like*			←		←	
*Hemocyte protease-2*		→				
*Insect intestinal lipase-6*					←	
*Lipase-1*		→	←		←	
*Pancreatic lipase-like*					→	
*Serine protease*	←					
*Trypsin-like*	←					←
*Zinc carboxypeptidase A1*						←
**detoxification/metabolism**						
*Antennal esterase CXE14*			←			
*Aquaporin*				→		
*Cytochrome P450*		→				
*Dicarbonyl/L-xylulose reductase*						←
*Esterase FE4*						←
*Farnesyl diphosphate synthase*				→		
*Fatty acid binding protein*			←			
*Similar to carboxylesterase*		←				
*Sugar transporter*			←			
*Putative galactose UDP 4-epimerase*			→			
**stress response**						
*eIF-4E-1*						→
*HSP 20*.*8*						→
*HSP 23*.*7*		→				
*HSP25*.*4*						→
**signaling**						
*Calreticulin*			→			
*Coiled-coil & C2 domain-containing 2A*					→	
*Ecdysteroid-22 kinase*				→		
*Famesoic acid O-methyltransferase*			→			
*FK506-binding protein precursor*			→			
*GTP*:*AMP phosphotransferase*		←				
*GTP-binding protein RAB2*				→		
*Inorganic phosphate transporter 1*			←			
*Juvenile hormone diol kinase*		→			→	
*Neuropeptide-like 4E (NPLP4E)*						←
*Palmityl transferase (P260)*				←	←	
*Protein kinase C inhibitor*			→			
*Troponin-C*					→	
**immune response**						
*Antitrypsin precursor*		←				
*Insulin-binding protein 2*		→			→	→
*Peptidoglycan recognition protein LB-like*						→
*Serpin-5*		→				
*Serpin-28*				→		
*Thioredoxin-like*				→		
*Tumor necrosis factor 13 (TNFSF13)*				→		
**RNAi**						
*Argonaute-2*						→
*Dicer-2*						→
**apoptosis**						
*Carboxypeptidase B*						→
*Caspase-8*						→
*Inhibitor-of-apoptosis*	←					
*Programmed cell death protein 5*			→			
*Putative apoptosis inhibitor 5*				←		
*30K protein 26*					←	
**ubiquitin-proteasome**						
*Goliath E3 ubiquitin ligase*				→		
*Ubiquitin-conjugating enzyme E2 J1-like*				→		
**midgut remodeling—cuticle**						
*CPH43*						→
*Urbain precursor*				→		
*37 kDa protease precursor*				→		
**iron metabolism**						
*Ferritin*	→					
*Transferrin*				→	→	
**ribosomal proteins**						
*L11*	→					
*P0*		→				
**unknown**						
*LOC101735726*						←
*Protein KGM_04122*					←	
*Protein KGM16290*					←	
*Similar to CG10527*					→	
*Similar to CG8927*				→		
*Unknown secreted protein*			→			
*30 kDa protein*					→	

Only genes confirmed by qRT-PCR (more than 2-fold difference in response) are shown. Induced and repressed genes are marked with up and down arrows respectively. Empty cells indicate that the particular gene was not tested by qRT-PCR for differential expression in the particular study. Abbreviations: SH: subtractive hybridization, MA: microarray, DS: deep sequencing. P50, 4008 and Daizo refer to silkworm strains.

The differences in transcriptional response among the four studies can be explained by the use of different silkworm strains, stage of infection and time of collection of infected samples. Gao et al. [16] compared the transcriptional response to BmCPV between a relatively resistant (p50) and a susceptible (4008) silkworm strain and found important differences in the response to virus infection. Of interest, it was observed that *ibp2* gene was induced at high levels only in the resistant strain. Therefore it was considered as a gene involved in resistance against BmCPV. The observation of induction of *ibp2* during pathogenic infection in our study suggests the existence of an active antiviral resistance mechanism during persistent infection with BmCPV.

### Transcriptional response of RNAi-related genes to infection with BmCPV in *B*. *mori*

As outlined in the introduction, a second goal in our study was to assess the RNAi response during a persistent and pathogenic infection with BmCPV since this response did not receive much attention in previous studies. Therefore, the transcriptional response of RNAi-related genes in the host tissues was examined, while the production of vsRNAs was also investigated.

It is well known that in *Drosophila* and mosquitoes, the siRNA pathway (and piRNA pathway in mosquitoes) can play an active role in the defense against exogenous invading single-stranded and double-stranded RNAs [63]. Due to the fact that the BmCPV genome consists of 10 segments of dsRNA, it was speculated that it could act as a target for the RNAi mechanism during the establishment of pathogenic BmCPV infection in *Bombyx* larvae. Therefore, it would be interesting to investigate whether pathogenic infection could up-regulate the expression of RNAi-related genes as an antiviral response mechanism during pathogenic infection.

**Fig 6:**
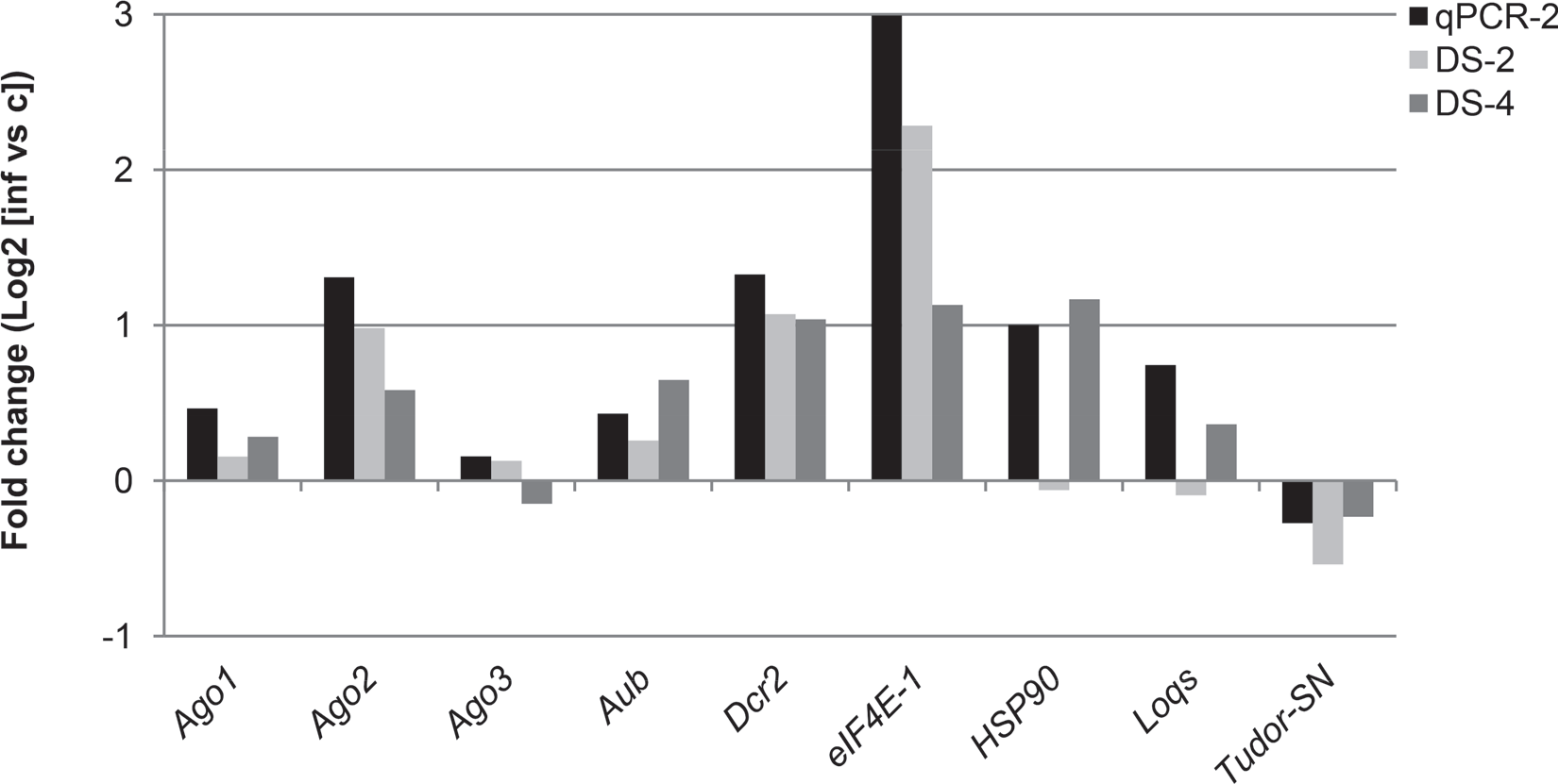
Expression levels of RNAi-related genes from pathogenically infected midguts as analyzed by deep sequencing and validated by qRT-PCR. Indicated is the fold change in expression of selected genes between persistent and pathogenic infection as obtained by qRT-PCR on 2^nd^ instar samples (qPCR-2) or by deep sequencing on both 2^nd^ and 4^th^ instar samples (DS-2 and DS-4, respectively). Please note that fold changes are expressed as log2 values (a 2-fold up- or down-regulation corresponds to a log2 value of 1 or -1, respectively). See Table 5 and [64] for further explanation on gene identity and function.

Following application of the BLASTP algorithm in the NCBI database on the *B*. *mori* genome, as well as use of the BLAST tool in the www.silkdb.org database, homologs (annotated or not) of several RNAi-related genes originally implicated in the RNAi process in other organisms were identified in the silkworm’s genome (for a discussion of different categories of RNAi-related genes, see [64]). Deep sequencing data were then analyzed to pinpoint alterations of the expression of RNAi-related genes following a pathogenic infection with BmCPV. The expression of several core RNAi genes and additional RNAi-related factors (Table 4) was further confirmed by qRT-PCR in 2^nd^ instar midgut cDNA samples (Fig. 6; S6 Fig.).

**Table 4:** Relative RPKMs of genes coding for core RNAi factors.

Core RNAi factors	2inf vs 2c RPKM	4inf vs 4c RPKM
**miRNA pathway**		
Pasha (part 1)	0.86	1.19
Pasha (part 2)	0.81	1.18
Dicer-1-like (part 1)	0.97	0.96
Dicer-1-like (part 2)	1.03	0.91
Loquacious	0.94	1.28
Argonaute 1	1.12	1.20
**siRNA pathway**		
Dicer 2 (part 1)	**2.20**	**2.08**
Dicer 2 (part 2)	**2.01**	**1.97**
R2D2	1.20	1.06
Argonaute 2	**1.98**	1.48
**piRNA pathway**		
Argonaute 3	1.10	0.89
Piwi/ Aubergine	1.20	**1.54**

Ratios of RPKM values from pathogenically versus persistently infected midgut tissue of 2^nd^ and 4^th^ instar larvae obtained by deep sequencing analysis (2c, 2inf, 4c and 4inf samples) are presented for selected genes belonging to the miRNA, siRNA and piRNA pathways. Genes presenting higher than 1.5-fold up- or down-regulation are marked with bold letters.

Three genes of the Argonaute family (i.e. *Ago1*, *Ago2* and *Ago3* that represent the miRNA, siRNA and piRNA pathway, respectively), as well as *Dcr2* from the siRNA pathway, are considered as core RNAi genes in *B*. *mori*, since inhibition of their expression by use of the “RNAi-of- the RNAi” method caused a significant impediment of the silencing potency in the silkworm-derived Bm5 cells [65]. In addition, several other core RNAi genes, such as *Aubergine* (*SIWI;* piRNA pathway) and *Loquacious* (dsRNA binding protein; miRNA pathway), were also analyzed in the deep sequencing database and by qRT-PCR (Table 4; Fig. 6; S6 Fig.).

Regarding the siRNA pathway, the genes *Ago2* and *Dcr2*, which are well known to be responsible for the defense against exogenous dsRNAs in *Drosophila*, showed a ~2-fold up-regulation in expression during the pathogenic infection (Table 4; Fig. 6; S6 Fig.). The piRNA pathway gene *Aubergine* was found to be 1.5-fold up-regulated in the 4^th^ instar animals after feeding with polyhedra; however, this result was not confirmed for the 2^nd^ instar stage. Also for *Loquacious* (miRNA pathway), no important differential expression between the two types of infection was observed (Table 4; Fig. 6; S6 Fig.).

Apart from the core RNAi components, additional RNAi-related genes were selected [64]. Tudor staphylococcus / micrococcal nuclease (Tudor-SN) and eukaryotic initiation factor-4E1 (eIF-4E1) were recently shown to constitute core factors for the formation of stress granules (SGs) in silkworm BmN4 cells, while they possibly also interact with Ago1 and Ago2 proteins of the miRNA and the siRNA pathway, respectively [66]. Tudor-SN expression was not notably altered after a pathogenic infection with BmCPV (Table 5; Fig. 6; S6 Fig.). Interestingly, however, the expression level of *eIF-4E1* was up-regulated during the pathogenic infection (from 2- to 8-fold, depending on the technique used and the sample; Table 5; Fig. 6; S6 Fig.). The increase of the relative mRNA level could be due to the general stress state caused by the infection, or due to a special role of *eIF-4E1* in the midgut response.

**Table 5:** Relative RPKMs of RNAi-related genes.

RNAi factors	Closest homolog in B. mori	GenBank Accession Number	Gene ID	2inf vs 2c RPKM	4inf vs 4c RPKM
**Intracellular auxiliary factors**					
Arginine methyltransferase PRMT5 (dPRMT5, also called capsuleen or dart5)	protein arginine N-methyltransferase 5-like isoform X1 or X2 (predicted)	XP_004928553.1/ XP_004928554.1	BGIBMGA007842	1.11	1.46
Armitage	probable RNA helicase armi-like (predicted)*	XP_004933936.1	BGIBMGA005056	1.13	0.95
ATP-dependent RNA helicase Belle	ATP-dependent RNA helicase DDX3X isoform X1 or X2 (predicted)	XP_004924684.1/ XP_004924685.1	BGIBMGA007594	1.05	1.26
Clp1 homolog (kinase)	protein CLP1 homolog (predicted)	XP_004923397.1	BGIBMGA004784	1.03	1.20
Drosophila homolog of p68 RNA helicase	DEAD box polypeptide 5 isoform 1 or 2	NP_001037582.1/ NP_001166829.1	BGIBMGA011746	1.31	1.42
	DEAD box polypeptide 5 isoform 1 or 2	NP_001037582.1/ NP_001166829.1	BGIBMGA011754	1.12	1.15
	DEAD box polypeptide 5 isoform 1 or 2	NP_001037582.1/ NP_001166829.1	BGIBMGA011965	1.05	1.22
	DEAD box polypeptide 5 isoform 1 or 2	NP_001037582.1/ NP_001166829.1	BGIBMGA012013	0.88	0.87
Elp-1	elongator complex protein 1	NP_001182006.1	BGIBMGA001538	0.94	0.97
Eukaryotic initiation factor 4E-1	eukaryotic initiation factor 4E-1	NP_001091832.1	BGIBMGA012675	**4.89**	**2.16**
Gawky CG31992-PA (similar)	trinucleotide repeat-containing gene 6B protein-like (predicted)/ protein Gawky-like (predicted)	XP_004922030.1/ XP_004922029.1	BGIBMGA005589	1.12	0.90
Gemin 3 homolog	probable ATP-dependent RNA helicase DDX20-like (predicted)	XP_004931522.1	BGIBMGA011804	0.77	1.41
HEN1	small RNA 2'-O-methyltransferase-like (predicted)	XP_004928786.1	BGIBMGA007679	1.21	0.79
Homeless (spindle-E)	probable ATP-dependent RNA helicase spindle-E-like (predicted)	XP_004924494.1	BGIBMGA004949	0.92	1.43
Maelstrom	protein maelstrom homolog (predicted)	XP_004928380.1	BGIBMGA008424	1.08	1.32
Putative translin-associated factor X	translin-associated protein X-like (predicted)	XP_004927402.1	BGIBMGA004644	nd	nd
Staufen	maternal effect protein staufen-like (predicted)	XP_004932448.1	BGIBMGA002695	1.12	0.88
Translin	translin	ABD36365.1	BGIBMGA009726	1.07	1.38
Tudor-SN	tudor staphylococcus/micrococcal nuclease	NP_001182009.1	BGIBMGA013328	0.69	0.85
Vasa intronic gene (VIG)	plasminogen activator inhibitor 1 RNA-binding protein-like	NP_001266293.1	BGIBMGA006704	1.07	0.86
**DsRNA uptake**					
CG4966 (orthologous to the Hermansky- Pudlak Syndrome 4, HPS4)	uncharacterized protein LOC101743016 (predicted)*	XP_004923697.1	BGIBMGA009850	1.16	**0.51**
Eater	neurogenic locus notch homolog protein 1-like (predicted) *	XP_004931260.1	BGIBMGA002383	1.33	0.79
FBX011 ortholog	F-box only protein 11-like isoform X1 or X2 (predicted)	XP_004924574.1/ XP_004924575.1	BGIBMGA005003	1.09	1.12
Scavenger receptor SR-C-like protein	scavenger receptor type C precursor	NP_001128387.1	BGIBMGA004577	**2.15**	**0.33**
Sid-1-like protein 1	sid-1-related gene 1 precursor/ low quality protein: SID1 transmembrane family member 1 (predicted)	NP_001106735.1 / XP_004930735.1	BGIBMGA011251	1.14	0.95
	sid-1-related gene 1 precursor	NP_001106735.1	BGIBMGA011160	0.92	0.87
	sid-1-related gene 1 precursor	NP_001106735.1	BGIBMGA011161	0.71	1.15
	low quality protein: SID1 transmembrane family member 1 (predicted)	XP_004930735.1	BGIBMGA011250	0.99	1.08
Sid-1-like protein 2	sid-1-like protein 2	BAF95807.1	BGIBMGA011251	1.14	0.95
Sid-1-like protein 3	sid-1-related gene 3 precursor	NP_001106736.1	BGIBMGA005847	0.93	0.84
**Antiviral RNAi**					
Ars2	low quality protein: serrate RNA effector molecule homolog (predicted)	XP_004930189.1	BGIBMGA003480	0.94	1.08
CG4572	venom serine carboxypeptidase-like (predicted)	XP_004929002.1	BGIBMGA013085	1.01	**1.85**
Egghead	glycosyltransferase precursor	NP_001243979.1	BGIBMGA001169	1.34	1.52
NinaC	myosin-IIIa-like (predicted)	XP_004930229.1	BGIBMGA003454	1.46	0.67
**Nucleases**					
dsRNase	alkaline nuclease precursor	NP_001091744.1	BGIBMGA001173	**0.55**	0.85
Exosome	PM-Scl autoantigen-like protein	NP_001108472.1	BGIBMGA000626	1.34	0.77
Nibbler	probable exonuclease mut-7 homolog (predicted)	XP_004931095.1	BGIBMGA002947	0.84	0.94
Poly(A) polymerase (Pla1 homolog *Schizosaccharomyces*)	poly(A) polymerase gamma-like isoform X1 (predicted)	XP_004923145.1	BGIBMGA000084	1.29	1.02
Sdn1-like (small RNA-degrading nuclease 1)	putative RNA exonuclease NEF-sp-like (predicted)	XP_004931480.1	BGIBMGA011782	0.84	1.31
**Other factors**					
Exportin-5	exportin-5-like (predicted)	XP_004928015.1	BGIBMGA001123	0.92	1.02
HSP90	HSP90, partial	AEB39782.1	BGIBMGA004612	0.96	**2.24**

Ratios of RPKM values from pathogenically versus persistently infected midgut tissue of 2^nd^ and 4^th^ instar larvae obtained by deep sequencing analysis (2c, 2inf, 4c and 4inf samples) are presented for selected genes with a role in the RNAi mechanism. Listed are intracellular auxiliary factors, dsRNA uptake genes, antiviral RNAi genes, nucleases and unclassified factors [64]. Genes presenting higher than 1.5-fold up- or down-regulation are marked with bold letters. Abbreviation: nd: not detected.

Heat shock protein 90 (HSP90) is an important factor that lately has emerged as a key piRNA pathway regulator. HSP90 was shown to take part in the piRNA pathway in BmN4 cells, more specifically in the recruitment of precursor piRNA molecules by the PIWI proteins [67, 68]. Although deep sequencing analysis showed a >2-fold up-regulation of *HSP90* in the 4^th^ instar library of pathogenically infected midgut tissue, this was not confirmed in the respective 2^nd^ instar sample (Table 5; Fig. 6; S6 Fig.). These data indicate that the role of HSP90 is relatively minor in this type of infection, in sharp contrast to other HSPs as shown in Tables 1–3.

Regarding genes involved in dsRNA uptake, *CG4572* and *HPS4* only showed clear differential expression between pathogenic and persistent infection in the 4^th^ instar animals. Also for the moderately expressed *scavenger receptor-C* (*SR-C*), no clear differential expression was observed (Table 5).

### Alteration in immune gene expression after infection with BmCPV

Genes belonging to innate immunity pathways were identified and analyzed regarding their possible differential expression upon infection (Table 6). Although differences were observed, these could often not be considered as biologically important because of low expression levels (RPKMs). However, a few exceptions were noted, such as the 2-fold down-regulation of *Toll6* and of one *Attacin1* homologue, as well as the 2-fold up-regulation of *beta-1*,*3-glucan recognition protein 2* gene in pathogenically infected larvae. The expression of *cecropins* (A, B and E) also tended to be up-regulated during the pathogenic infection. On the other hand, *Toll9-1*, which was previously found to be down-regulated by exogenous dsRNA application [69], was not differentially expressed in any of the two pathogenically infected midgut samples.

**Table 6:** Relative RPKMs of innate immune genes.

Description	Gene ID	2inf RPKM	2c RPKM	4inf RPKM	4c RPKM	inf Means	c Means	2inf vs 2c RPKM	4inf vs 4c RPKM
**Toll pathway**									
MYD88	BGIBMGA002869	9.68	8.97	7.46	8.64	8.57	8.80	1.08	0.86
RelA	BGIBMGA010496	1.49	1.35	0.89	1.04	1.19	1.20	1.10	0.85
RelB	BGIBMGA010497	1.83	1.38	1.24	1.21	1.54	1.30	1.32	1.03
Toll3-1	BGIBMGA014370	2.53	2.75	2.84	2.65	2.68	2.70	0.92	1.07
Toll3-2	BGIBMGA014373	23.77	25.15	25.60	36.49	24.68	30.82	0.95	0.70
Toll3-2	BGIBMGA014372	0.17	0.24	0.05	0.19	0.11	0.22	0.70	**0.24**
Toll3-3	BGIBMGA010304	0.04	0.05	0.02	0.04	0.03	0.05	0.75	**0.44**
Toll6	BGIBMGA011084	0.68	1.27	0.06	0.11	0.37	0.69	**0.54**	**0.53**
Toll7-1/7-2	BGIBMGA011037	1.56	1.12	0.37	0.20	0.97	0.66	1.40	**1.90**
Toll7-2	BGIBMGA011034	3.53	3.75	2.09	2.46	2.81	3.10	0.94	0.85
Toll7-3	BGIBMGA011038	0.64	0.32	0.00	0.02	0.32	0.17	**1.98**	0.00
Toll8	BGIBMGA011085	1.25	0.77	1.31	1.18	1.28	0.97	1.63	1.12
Toll9-1	BGIBMGA011216	17.21	16.50	20.90	30.49	19.05	23.49	1.04	0.69
Toll9-2	BGIBMGA008840	0.53	0.55	0.44	1.25	0.48	0.90	0.96	**0.35**
Toll10-2	BGIBMGA011025	1.85	2.28	0.50	0.28	1.17	1.28	0.81	**1.75**
Toll10-3	BGIBMGA011082	6.02	6.30	2.76	1.38	4.39	3.84	0.96	**2.00**
Toll12	BGIBMGA006244	0.81	0.96	0.29	0.10	0.55	0.53	0.84	**2.96**
Tube	BGIBMGA002494	11.09	10.93	8.48	9.71	9.78	10.32	1.01	0.87
**Imd pathway**									
Ikkβ	BGIBMGA008389	21.76	22.15	15.64	18.38	18.70	20.26	0.98	0.85
Imd	BGIBMGA003655	2.42	2.30	1.75	2.20	2.08	2.25	1.05	0.79
Relish1	BGIBMGA002465	6.44	5.83	4.27	4.10	5.36	4.97	1.10	1.04
Relish1	BGIBMGA002464	1.16	1.31	3.01	3.74	2.08	2.53	0.88	0.80
Tak1	BGIBMGA008980	7.20	6.79	4.51	5.21	5.86	6.00	1.06	0.87
**PPO pathway**									
Prophenoloxidase activating enzyme	BGIBMGA013746	0.25	0.12	0.17	0.39	0.21	0.26	**2.11**	**0.44**
Serine protease inhibitor	BGIBMGA009047	11.71	11.48	32.27	27.01	21.99	19.25	1.02	1.19
Serine protease inhibitor 6	BGIBMGA007729	1.57	1.52	1.03	0.48	1.30	1.00	1.03	**2.16**
**Pattern recognition receptor**									
C-type lectin 10	BGIBMGA006768	1.40	2.27	0.25	0.12	0.83	1.19	**0.62**	**2.14**
C-type lectin 21	BGIBMGA002288	0.23	0.29	0.23	0.11	0.23	0.20	0.79	**2.14**
Beta-1,3-glucan recognition protein 2	BGIBMGA011609	3.28	1.72	1.26	0.46	2.27	1.09	**1.91**	**2.74**
Peptidoglycan recognition protein (PGRP-L2)	BGIBMGA000584	2.92	2.67	1.81	2.58	2.36	2.63	1.09	0.70
Peptidoglycan recognition protein S6	BGIBMGA012866	0.52	0.29	0.38	0.29	0.45	0.29	**1.83**	1.32
**Antimicrobial peptide**									
Attacin1	BGIBMGA002739	0.72	0.19	0.27	0.00	0.50	0.10	**3.77**	na
Attacin1	BGIBMGA002747	0.86	2.29	0.14	0.31	0.50	1.30	**0.38**	**0.44**
CecropinA, CecropinB	BGIBMGA000024	0.22	0.22	0.11	0.00	0.16	0.11	1.00	na
	BGIBMGA000036	1.18	0.78	0.00	0.00	0.59	0.39	**1.51**	na
	BGIBMGA000037	6.12	3.54	0.00	0.00	3.06	1.77	**1.73**	na
	BGIBMGA000021	23.38	8.49	0.00	0.00	11.69	4.24	**2.75**	na
	BGIBMGA000038	2.15	0.78	0.00	0.00	1.07	0.39	**2.76**	na
	BGIBMGA000023	0.00	0.52	0.00	0.00	0.00	0.26	0.00	na
CecropinA, CecropinE	BGIBMGA006280	3.16	1.90	2.45	0.22	2.80	1.06	**1.67**	**11.18**
Defensin	BGIBMGA014360	0.00	0.16	0.08	0.11	0.04	0.14	**0.00**	**0.66**
Gloverin	BGIBMGA013866	0.00	0.32	0.00	0.00	0.00	0.16	**0.00**	na
	BGIBMGA013864	5.89	5.69	5.98	7.29	5.94	6.49	1.04	0.82
	BGIBMGA013863	0.17	0.37	0.21	0.19	0.19	0.28	**0.46**	1.10
Gloverin 2	BGIBMGA005658	1.52	1.53	12.97	8.28	7.25	4.91	0.99	**1.57**
Gloverin 3	BGIBMGA013803	0.06	0.08	0.17	0.13	0.11	0.10	0.75	1.32
Gloverin 4	BGIBMGA013865	0.16	0.14	0.05	0.11	0.10	0.13	1.13	**0.44**
Hemolin	BGIBMGA008736	0.11	0.44	0.22	0.31	0.16	0.37	**0.26**	0.69
Lebocin 1/2/3/4	BGIBMGA006775	0.22	1.15	1.24	0.79	0.73	0.97	**0.19**	**1.58**
Lysozyme	BGIBMGA007458	0.63	0.66	0.17	0.43	0.40	0.55	0.95	**0.39**
Moricin	BGIBMGA011495	0.67	0.44	0.11	0.33	0.39	0.39	**1.51**	**0.33**
**JAK/STAT pathway**									
STAT	BGIBMGA001739	4.04	3.35	4.10	4.36	4.07	3.86	1.21	0.94

Ratios of RPKM values from pathogenically versus persistently infected midgut tissue of 2^nd^ and 4^th^ instar larvae obtained by deep sequencing analysis (2c, 2inf, 4c and 4inf samples) are presented for selected genes involved in innate immunity pathways. Listed are genes belonging to Toll, Imd, PPO and JAK/STAT pathways, as well as genes encoding pattern recognition receptors and antimicrobial peptides. Genes presenting higher than 1.5-fold up- or down-regulation are marked with bold letters. Abbreviation: na: not applicable.

### Detection and preliminary analysis of viral small RNAs (vsRNAs)

Deep sequencing was also used for the analysis of the small RNAs in samples of persistently and pathogenically infected larvae. Filtering of the small RNAs for reads that map to the BmCPV genome resulted in a total of 4,487,417 reads for all 4 samples. When the number of reads of the vsRNAs was plotted against their length, a clear peak of 20 nt vsRNAs equally distributed between both genomic dsRNA strands appeared in all 4 samples. As expected, vsRNA reads were highly abundant in pathogenically infected midguts, while for persistently infected samples their abundance was notably low. The peak of 20 nt corresponds to 749,372 and 2,079,497 reads for the 2inf and 4inf samples, respectively, while for the control samples 2c and 4c the small RNA counts were 324 and 481, respectively (Fig. 2).

These data clearly indicate that the RNAi machinery is responding to BmCPV infection and that the abundance of produced vsRNAs is correlated with the severity of the infection. Further analysis of the vsRNA data such as the mapping of the vsRNAs to viral dsRNA genome segments to detect hot-spots of small RNA production and to identify differences in vsRNAs between persistently and pathogenically infected animals is currently being carried out (manuscript in preparation). The observation that the vsRNAs mapped equally to sense and antisense strands of the dsRNA genome (Fig. 2) strongly indicates that the dsRNA genome segments, rather than structured dsRNA regions of viral (sense) mRNAs, are the source for production of vsRNAs by Dicer enzymes. Further studies are required to establish the functionality of the vsRNAs and to investigate whether differences in activity exist between vsRNAs produced in persistently versus pathogenically infected animals as well as between different regions of the viral dsRNA genome (cold-spots versus hot-spots).

## Conclusions

In this work, the discovery of persistent BmCPV infection of our silkworm laboratory colony provided an opportunity to compare the transcriptional response to pathogenic infection with that occurring in non-persistently infected larvae, as described in the literature. Our conclusions can be summarized as follows:

1. The transcriptional response against pathogenic BmCPV infection is complex and suggests the involvement of several mechanisms, including RNAi.
2. Pre-existing persistent infection does not profoundly affect the antiviral response against pathogenic infection with the same virus, as documented by our analysis in comparison to previously reported studies.
3. Detection of vsRNAs by deep sequencing indicates the activation of the RNAi response to both persistent and pathogenic infection of BmCPV.

## Supporting Information

S1 Dataset Expression data of all genes detected in the database obtained from deep sequencing of persistently and pathogenically infected midgut tissue of 2^nd^ and 4^th^ instar larvae (2c, 2inf, 4c and 4inf libraries). Shown are RPKMs for each gene in each individual library (after trimming), as well as their average RPKMs in persistently and pathogenically infected samples. Total (not normalized) reads for each gene are also shown. The last two columns show the differential expression in pathogenically versus persistently infected midgut tissue for 2^nd^ and 4^th^ instar developmental stages.
(XLSX)

S2 Dataset Expression data of all genes that are differentially expressed following pathogenic infection in at least one of the two library pairs obtained by deep sequencing (2c/2inf and 4c/4inf pairs, corresponding to persistent/pathogenic infection at 2^nd^ and 4^th^ instar stages). Shown are RPKMs for each gene in each individual library (after trimming), as well as their average RPKMs in persistently and pathogenically infected samples. Total (not normalized) reads for each gene are also shown. The last two columns show the differential expression in pathogenically versus persistently infected midgut tissue for 2^nd^ and 4^th^ instar developmental stages.
(XLSX)

S3 Dataset Expression data of the 308 genes that are differentially expressed following pathogenic infection in both library pairs obtained by deep sequencing (2c/2inf and 4c/4inf pairs, corresponding to persistent/pathogenic infection at 2^nd^ and 4^th^ instar stages). Shown are RPKMs for each gene in each individual library (after trimming), as well as their average RPKMs in persistently and pathogenically infected samples. Total (not normalized) reads for each gene are also shown. The last two columns show the differential expression in pathogenically versus persistently infected midgut tissue for 2^nd^ and 4^th^ instar developmental stages. Criteria for the selection of genes are described in detail in *Materials and Methods* section.
(XLSX)

S1 Fig *Bombyx mori* larvae persistently and pathogenically infected with BmCPV. Larvae of Daizo strain were orally infected with a high dose of BmCPV polyhedra at the 2^nd^ or the 4^th^ instar stage, or left untreated. The images show larvae 20 days (for 2^nd^ instar stage; 2c, 2inf) or 14 days (for 4^th^ instar stage; 4c, 4inf) after manipulation. Untreated larvae of the Daizo strain were persistently infected with BmCPV.
(TIF)

S2 Fig Detection of BmCPV polyhedra in pathogenically infected larvae. Cubic crystalline structures (viral polyhedra) were observed under the microscope in (a) midgut tissue, (b) body wall tissue and (c) hemolymph. Magnification factor: 40x.
(TIF)

S3 Fig Distribution of GO terms among highly differentially expressed genes in pathogenically infected larvae. All genes from S3 Dataset (corresponding to Fig. 2) having a GO annotation were categorized using GO tools in different classes representing biological process, molecular function and cellular component. Classification is shown at several levels of GO analysis.
(PDF)

S4 Fig Relative expression levels of selected genes from pathogenically infected midguts as determined by qRT-PCR. Expression of genes showing by deep sequencing significant levels of up-regulation during pathogenic infection was validated by qRT-PCR in midgut samples of persistently and pathogenically infected 2^nd^ instar larvae. The graphs depict mean values of expression normalized to the housekeeping gene *actin 3*, as measured for two biological and two technical replicates (+SE). For clarity, four different graphs (a-d) of relative mRNA levels are shown, in which genes with similar mRNA levels are grouped (see different scales in the graphs). High error bars obtained for expression of genes may reflect differences between samples in exact developmental stage or progression of viral infection. Expression of genes may be more strictly developmentally regulated or be more sensitive to progression of viral infection. See Table 2 for further explanation on gene identity and function.
(TIF)

S5 Fig The uncharacterized protein LOC101735726. Indicated are the conserved domains PIF1 and RecD as detected by the DELTA-BLAST algorithm. PIF1-like helicase domains are implicated in the regulation of telomerase activity during the cell cycle. RecD-like helicase domains are associated with functions in DNA replication, recombination and repair.
(TIF)

S6 Fig Relative expression levels of RNAi-related genes in pathogenically infected midguts as determined by qRT-PCR. Expression of several RNAi-related genes was analyzed by qRT-PCR in midgut samples of persistently and pathogenically infected 2^nd^ instar larvae. The graphs depict mean values of expression normalized to the housekeeping gene *actin 3*, as measured for two biological and two technical replicates (+SE). For clarity, four different graphs (a-d) of relative mRNA levels are shown, in which genes with similar mRNA levels are grouped (see different scales in the graphs). High error bars obtained for expression of genes may reflect differences between samples in exact developmental stage or progression of viral infection. Expression of genes may be more strictly developmentally regulated or more sensitive to progression of viral infection. See Table 6 and [64] for further explanation on gene identity and function.
(TIF)

S1 Table Sequences of primers used in qRT-PCR reactions for mRNA expression quantification. Genes are identified by their SilkDB ID (www.silkdb.org) and their GenBank Accession Number (www.ncbi.nlm.nih.gov/).
(DOCX)
