# Correction: Transcriptome Analysis of *Bombyx mori* Larval Midgut during Persistent and Pathogenic Cytoplasmic Polyhedrosis Virus Infection

**DOI:** 10.1371/journal.pone.0126452

**Published:** 2015-04-15

**Authors:** The PLOS ONE Staff

Table 3 is incorrect. The publisher apologizes for this error. Please see the corrected Table 3 here.

**Table 3:** 

Genes	SH (p50) ^[17]^	MA (p50) ^[18]^	DS (4008) ^[15]^	DS (4008) ^[16]^	DS (p50) ^[16]^	DS (Daizo) our study
**digestive enzymes (proteases, lipases, nucleases)**						
*Acidic lipase*			↓			
*Alkaline nuclease*	↑					
*Chymotrypsin-like*			↓		↓	
*Hemocyte protease-2*		↑				
*Insect intestinal lipase-6*					↓	
*Lipase-1*		↑	↓		↓	
*Pancreatic lipase-like*					↑	
*Serine protease*	↓					
*Trypsin-like*	↓					↓
*Zinc carboxypeptidase A1*						↓
**detoxification/metabolism**						
*Antennal esterase CXE14*			↓			
*Aquaporin*				↑		
*Cytochrome P450*		↑				
*Dicarbonyl/L-xylulose reductase*						↓
*Esterase FE4*						↓
*Farnesyl diphosphate synthase*				↑		
*Fatty acid binding protein*			↓			
*Similar to carboxylesterase*		↓				
*Sugar transporter*			↓			
*Putative galactose UDP 4-epimerase*			↑			
**stress response**						
*eIF-4E-1*						↑
*HSP 20*.*8*						↑
*HSP 23*.*7*		↑				
*HSP25*.*4*						↑
**signaling**						
*Calreticulin*			↑			
*Coiled-coil & C2 domain-containing 2A*					↑	
*Ecdysteroid-22 kinase*				↑		
*Famesoic acid O-methyltransferase*			↑			
*FK506-binding protein precursor*			↑			
*GTP*:*AMP phosphotransferase*		↓				
*GTP-binding protein RAB2*				↑		
*Inorganic phosphate transporter 1*			↓			
*Juvenile hormone diol kinase*		↑			↑	
*Neuropeptide-like 4E (NPLP4E)*						↓
*Palmityl transferase (P260)*				↓	↓	
*Protein kinase C inhibitor*			↑			
*Troponin-C*					↑	
**immune response**						
*Antitrypsin precursor*		↓				
*Insulin-binding protein 2*		↑			↑	↑
*Peptidoglycan recognition protein LB-like*						↑
*Serpin-5*		↑				
*Serpin-28*				↑		
*Thioredoxin-like*				↑		
*Tumor necrosis factor 13 (TNFSF13)*				↑		
**RNAi**						
*Argonaute-2*						↑
*Dicer-2*						↑
**apoptosis**						
*Carboxypeptidase B*						↑
*Caspase-8*						↑
*Inhibitor-of-apoptosis*	↓					
*Programmed cell death protein 5*			↑			
*Putative apoptosis inhibitor 5*				↓		
*30K protein 26*					↓	
**ubiquitin-proteasome**						
*Goliath E3 ubiquitin ligase*				↑		
*Ubiquitin-conjugating enzyme E2 J1-like*				↑		
**midgut remodeling—cuticle**						
*CPH43*						↑
*Urbain precursor*				↑		
*37 kDa protease precursor*				↑		
**iron metabolism**						
*Ferritin*	↑					
*Transferrin*				↑	↑	
**ribosomal proteins**						
*L11*	↑					
*P0*		↑				
**unknown**						
*LOC101735726*						↓
*Protein KGM_04122*					↓	
*Protein KGM16290*					↓	
*Similar to CG10527*					↑	
*Similar to CG8927*				↑		
*Unknown secreted protein*			↑			
*30 kDa protein*					↑	
