# Supplementary figures and images for: Transcriptome Analysis of Bombyx mori Larval Midgut during Persistent and Pathogenic Cytoplasmic Polyhedrosis Virus Infection
Source: PLoS One. 2015 Mar 27;10(3):e0121447. doi: 10.1371/journal.pone.0121447 (PMC4376736; doi:10.1371/journal.pone.0121447)

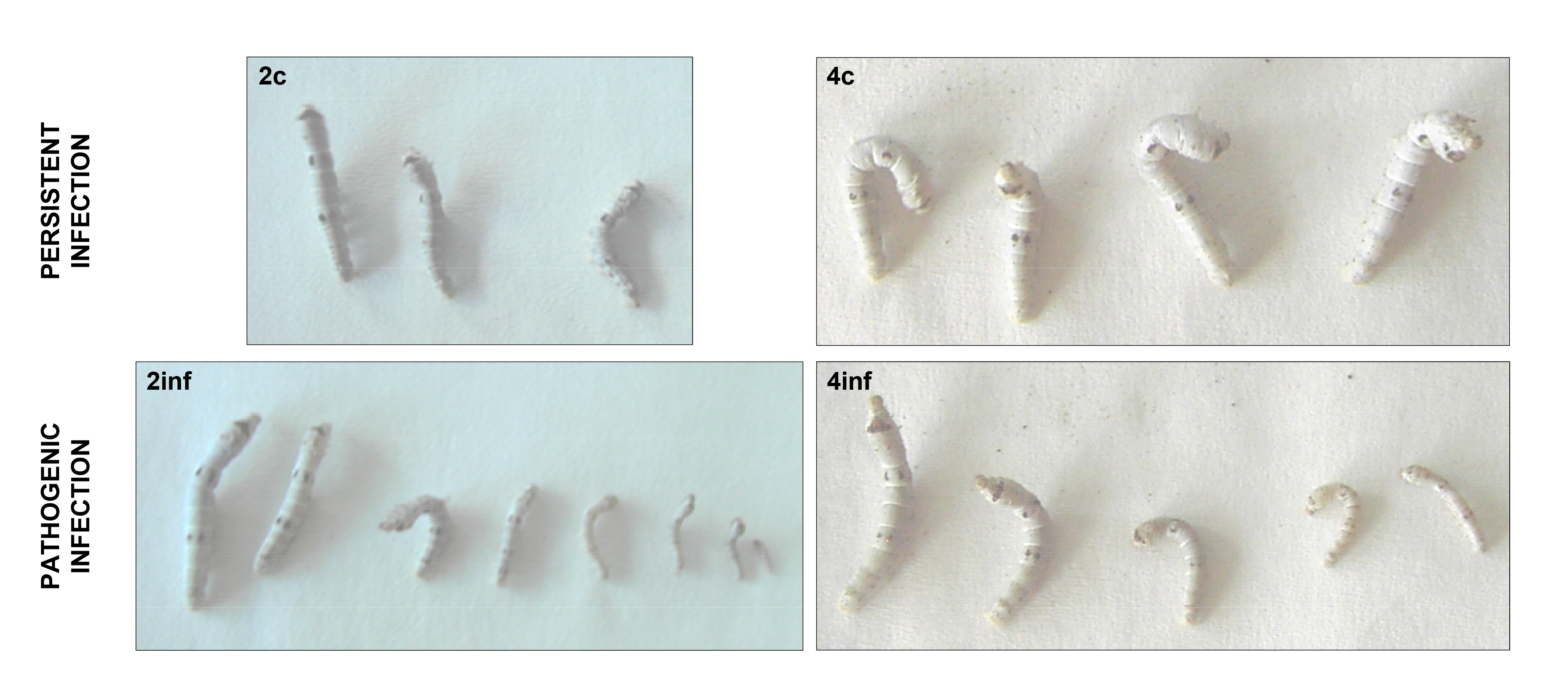

Supplement: S1 Fig — Larvae of Daizo strain were orally infected with a high dose of BmCPV polyhedra at the 2nd or the 4th instar stage, or left untreated. The images show larvae 20 days (for 2nd instar stage; 2c, 2inf) or 14 days (for 4th instar stage; 4c, 4inf) after manipulation. Untreated larvae of the Daizo strain were persistently infected with BmCPV. (TIF) [file pone.0121447.s004.tif]

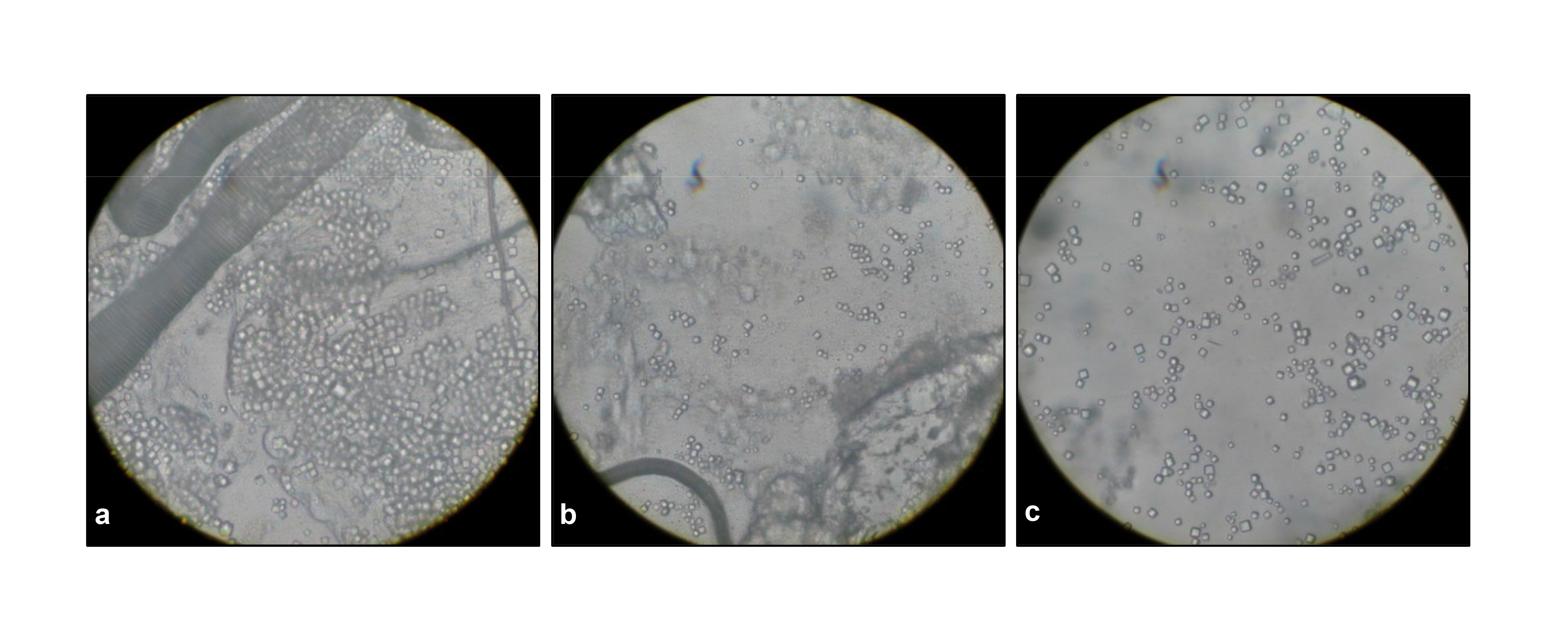

Supplement: S2 Fig — Cubic crystalline structures (viral polyhedra) were observed under the microscope in (a) midgut tissue, (b) body wall tissue and (c) hemolymph. Magnification factor: 40x. (TIF) [file pone.0121447.s005.tif]

Sequence distribution: molecular\_function

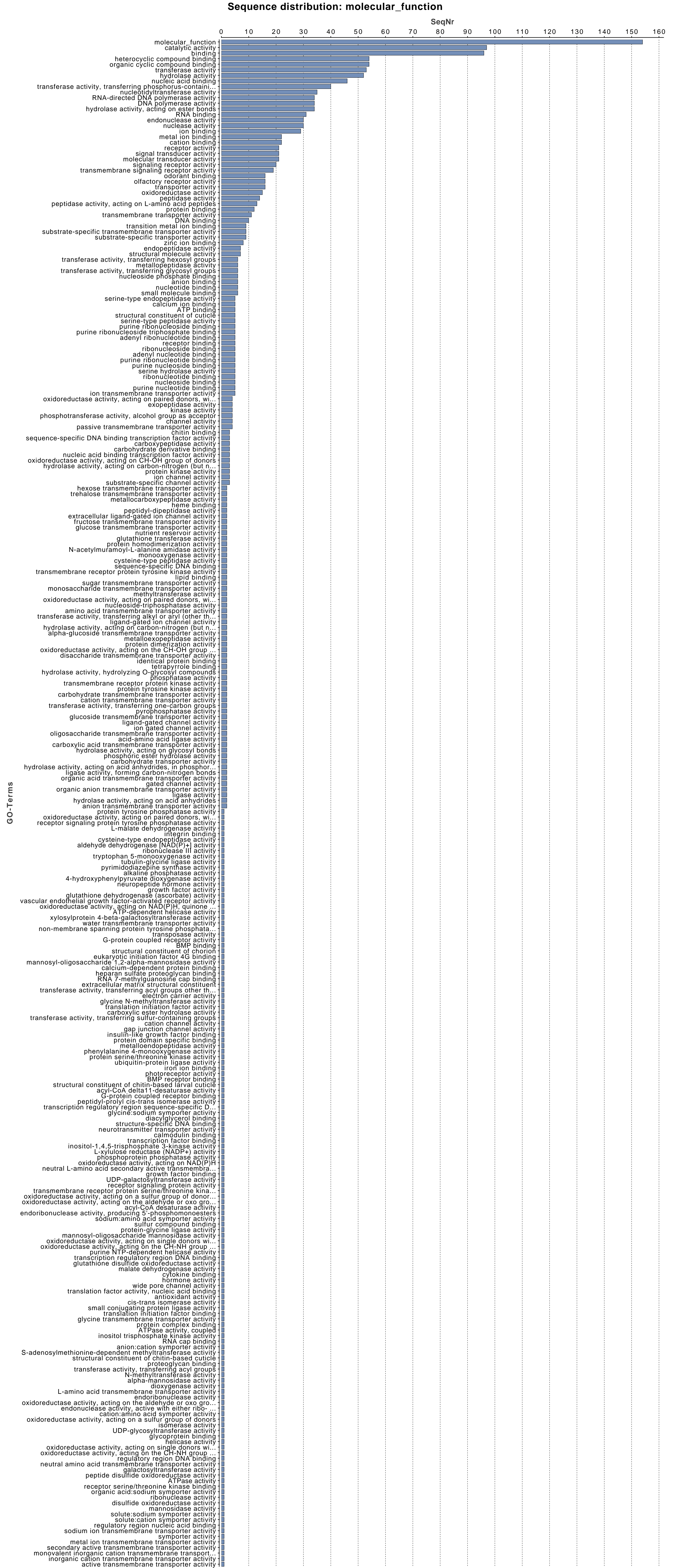

# Sequence distribution: cellular\_component

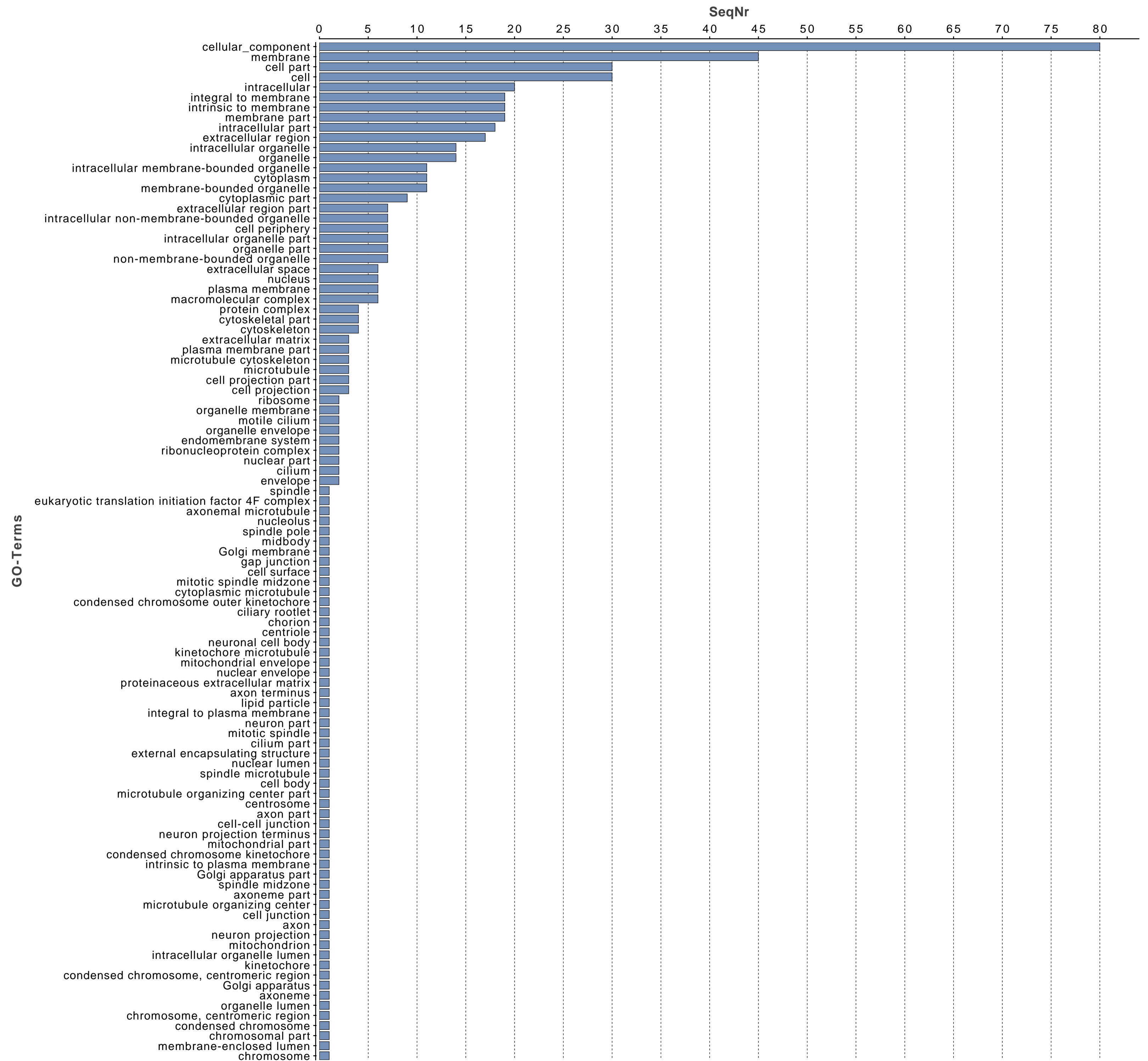

Supplement: S3 Fig — All genes from S3 Dataset (corresponding to Fig. 2) having a GO annotation were categorized using GO tools in different classes representing biological process, molecular function and cellular component. Classification is shown at several levels of GO analysis. (PDF) [file pone.0121447.s006.pdf]

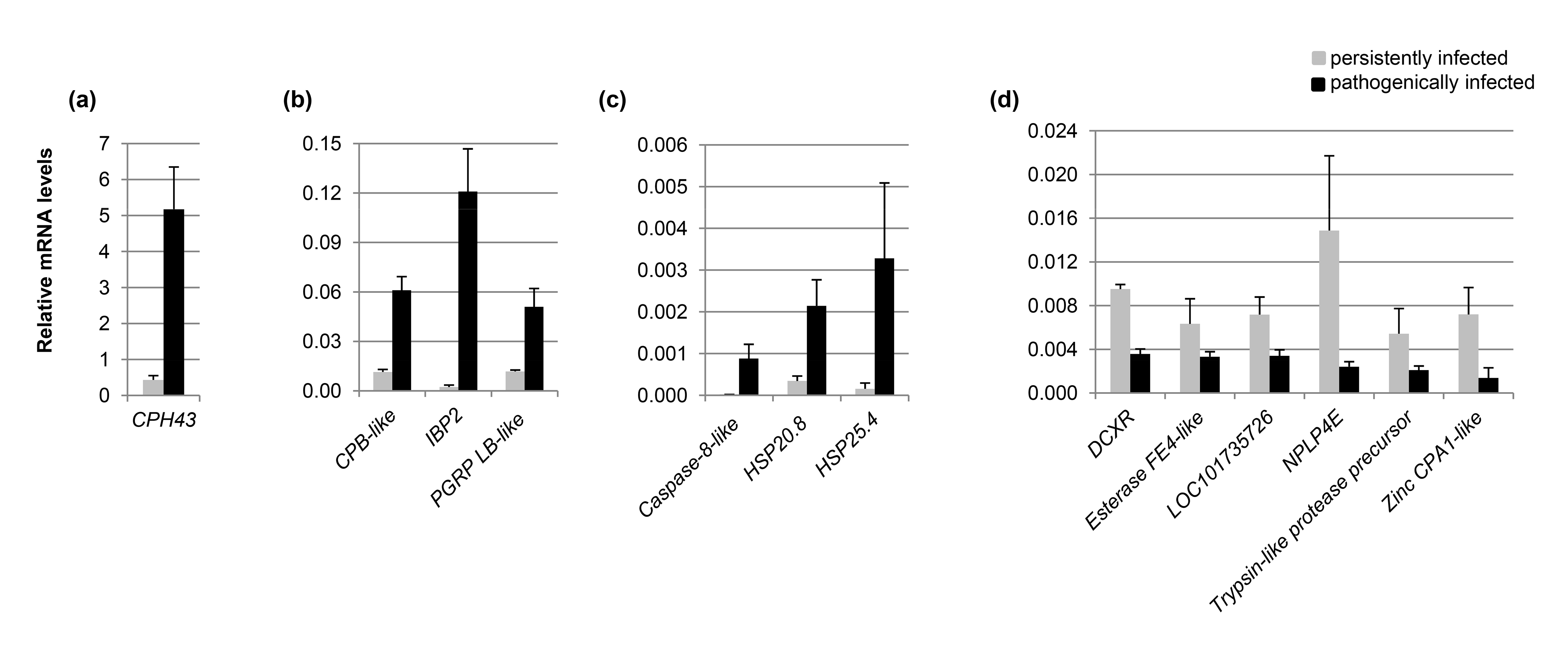

Supplement: S4 Fig — Expression of genes showing by deep sequencing significant levels of up-regulation during pathogenic infection was validated by qRT-PCR in midgut samples of persistently and pathogenically infected 2nd instar larvae. The graphs depict mean values of expression normalized to the housekeeping gene actin 3, as measured for two biological and two technical replicates (+SE). For clarity, four different graphs (a-d) of relative mRNA levels are shown, in which genes with similar mRNA levels are grouped (see different scales in the graphs). High error bars obtained for expression of genes may reflect differences between samples in exact developmental stage or progression of viral infection. Expression of genes may be more strictly developmentally regulated or be more sensitive to progression of viral infection. See Table 2 for further explanation on gene identity and function. (TIF) [file pone.0121447.s007.tif]

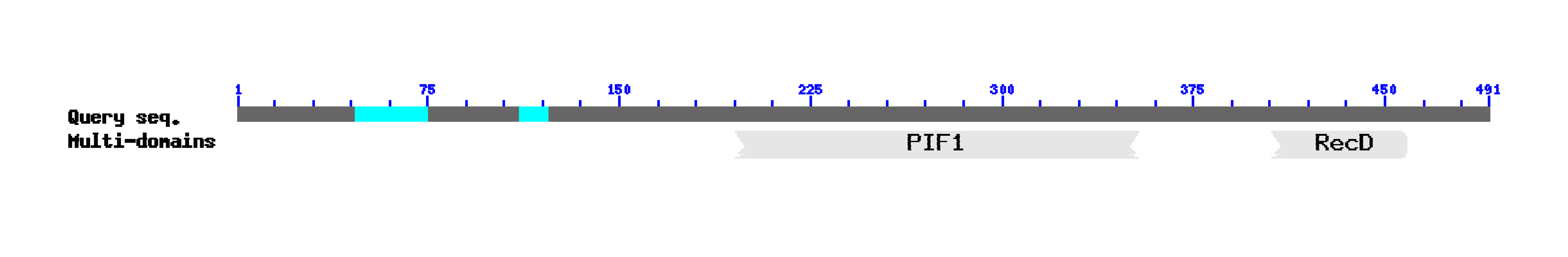

Supplement: S5 Fig — Indicated are the conserved domains PIF1 and RecD as detected by the DELTA-BLAST algorithm. PIF1-like helicase domains are implicated in the regulation of telomerase activity during the cell cycle. RecD-like helicase domains are associated with functions in DNA replication, recombination and repair. (TIF) [file pone.0121447.s008.tif]

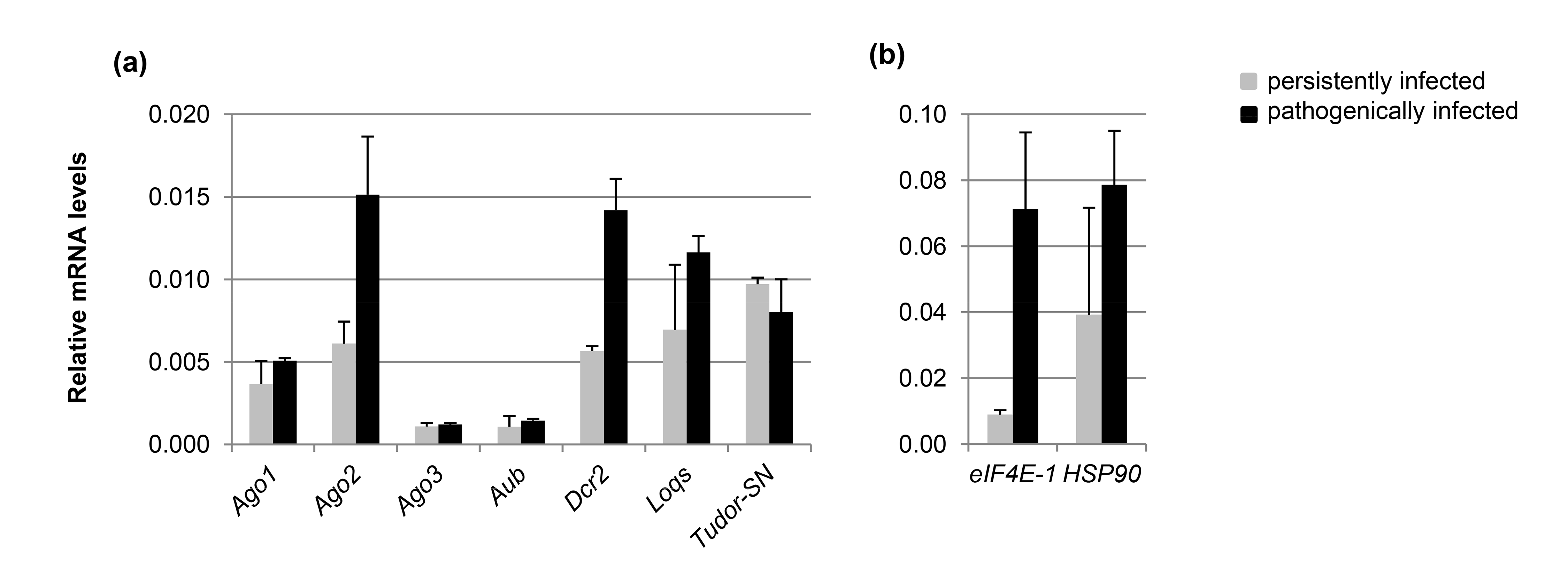

Supplement: S6 Fig — Expression of several RNAi-related genes was analyzed by qRT-PCR in midgut samples of persistently and pathogenically infected 2nd instar larvae. The graphs depict mean values of expression normalized to the housekeeping gene actin 3, as measured for two biological and two technical replicates (+SE). For clarity, four different graphs (a-d) of relative mRNA levels are shown, in which genes with similar mRNA levels are grouped (see different scales in the graphs). High error bars obtained for expression of genes may reflect differences between samples in exact developmental stage or progression of viral infection. Expression of genes may be more strictly developmentally regulated or more sensitive to progression of viral infection. See Table 6 and [64] for further explanation on gene identity and function. (TIF) [file pone.0121447.s009.tif]
