# Supplementary material for: Transcriptome Analysis of Bombyx mori Larval Midgut during Persistent and Pathogenic Cytoplasmic Polyhedrosis Virus Infection
Source: PLoS One. 2015 Mar 27;10(3):e0121447. doi: 10.1371/journal.pone.0121447 (PMC4376736; doi:10.1371/journal.pone.0121447)
Supplement: S1 Table — Genes are identified by their SilkDB ID (www.silkdb.org) and their GenBank Accession Number (www.ncbi.nlm.nih.gov/). (DOCX) [file pone.0121447.s010.docx]

| Gene | Primer | Primer sequence  (5'-3') | SilkDB ID | GenBank  Acc. Number |
| --- | --- | --- | --- | --- |
| *Actin A3*^[1]^ | Actin-qF | CCGTATGCAAAAGGAAATCA | BGIBMGA013945 | AB701689.1 |
|  | Actin-qR | TTGGAAGGTAGAGAGGGAGG |  |  |
| *Argonaute 1* | AGO1-1708F | GTTGTCGTCGTGTTACCAGGA | BGIBMGA003670 | NM_001102461.1 |
|  | AGO1-1784R | GCCATTCCCAATACTGTGTCA |  |  |
| *Argonaute 2*^[2]^ | AGO2-2349F | CGAAGCTAATGAATACCGGTCG | BGIBMGA010406 | AB206986.2 |
|  | AGO2-2453R | CGTGCAGCATACTCTTGTGCA |  |  |
| *Argonaute 3*^[2]^ | AGO3-2449F | TCGAGAGACGGCTACGACAAT | BGIBMGA009037 | NM_001104597.2 |
|  | AGO3-2541R | CTGCGAGACGATCAGGAAATC |  |  |
| *Aubergine* | Aub-2142F | TTTCAATATCGCGTCCGCC | BGIBMGA010644 | NM_001104596.2 |
|  | Aub-2225R | ACACCGTCGCGGTAAATGAAG |  |  |
| *Carboxypeptidase B-like* (predicted) | CarboxypeptidaseB-549F | AGAATGGATTTCCCCACCGA | BGIBMGA009487 | XM_004922757.1 |
|  | CarboxypeptidaseB-639R | CCAATCAAACCGGTCCAAAAG |  |  |
| *Caspase-8-like* (predicted) | Caspase8-3F | GCAATCTTCGAGTCCTGGACC | BGIBMGA008021 | XM_004922839.1 |
|  | Caspase8-73R | GGTTGCTGTGCAACACAAGG |  |  |
| *Dicarbonyl/ L-xylulose reductase* | LXReductase-406F | TCGCAAGCTTCTAAGGCAGC | BGIBMGA007259 | NM_001046967.1 |
|  | LXReductase-519R | TCGTATGCCCAAAGGTCCC |  |  |
| *Dicer 2*^[2]^ | Dcr2-4463F | AAATGACTGACCTGAGATCCGC | BGIBMGA011542 | AB566386.1 |
|  | Dcr2-4549R | AGCAGAGGTACTTGTGCAATCC | BGIBMGA011543 |  |
| *Esterase FE4-like* (predicted) | EsteraseFE4-68F | AGTTCGGTGGTGATCGTGAGA | BGIBMGA001963 | XM_004933446.1 |
|  | EsteraseFE4-152R | GCGGACGAATGCATAAGCA |  |  |
| *Eukaryotic initiation factor 4E-1* | eIF4E1-19F | CAACATGAGACGCGAGATGGT | BGIBMGA012675 | NM_001098362.1 |
|  | eIF4E1-99R | CCAAGTGTGCTCGAGAGGATG |  |  |
| *Heat shock protein 20.8* | HSP20.8-76F | CAAGATTTTGGATTGGCGCT | BGIBMGA004605 | FJ602772.1 |
|  | HSP20.8-205R | TTATGCTGGAACCGAGGTCAC |  |  |
| *Heat shock protein 25.4 precursor* | HSP25.4p-75F | TTGGCCGTATCACCATTACGA | BGIBMGA005781 | NM_001118903.1 |
|  | HSP25.4p-157R | CAAGGTTGGACCAAAGCGAA |  |  |
| *Heat shock protein 90* | HSP90-1768F | GGATGGTCTGCCAACATGGA | BGIBMGA004612 | NM_001043411.1 |
|  | HSP90-1844R | GCAGCCATGTATCCCATTGTG |  |  |
| *IBP2 insulin-related peptide binding protein* | IBP2-172F | CATCATACCACTGGCTCGCAA | BGIBMGA008146 | JX133233.1 |
|  | IBP2-270R | TTCCTCTATCGGTTCCCCGTT |  |  |
| *Loquacious* | Loqs-444F | CAATGGCAATGTTCCTGAAAC | BGIBMGA005395 | NM_001195079.1 |
|  | Loqs-531R | ACAACTCCTGAAGCCATCCAAC |  |  |
| *Neuropeptide-like precursor 4E* | NLP4E-126F | TTTCGCATCAGCCAAGCCT | BGIBMGA011764 | HQ386681.1 |
|  | NLP4E-254R | GCAACCGGAGAATATGCAGAG |  |  |
| *Peptidoglycan-recognition protein LB-like* (predicted) | PeptidoglycanRP-47F | AATCCGTCAAGGACCTGATCG | BGIBMGA012865 | XM_004928822.1 |
|  | PeptidoglycanRP-130R | TGACTTGTCTGTGTCCGACGA |  |  |
| *Putative cuticle protein CPH43* | CPH43-300F | ATTAGTGGTAAACGCGGTCGG | BGIBMGA005302 | GU070728.1 |
|  | CPH43-399R | TATTCCCGCTGAGCTGAAACC |  |  |
| *Trypsin-like protease precursor* | ProteaseP-488F | CTGACGGCAACCTCCACAAG | BGIBMGA010024 | NM_001099803.1 |
|  | ProteaseP-571R | CGACGCGATCATGACCCTT |  |  |
| *Tudor staphylococcus/ micrococcal nuclease (Tudor-SN)* | TudorSN-2017F | AACAGTCACCCATTGCCTGG | BGIBMGA013328 | NM_001195080.1 |
|  | TudorSN-2097R | CCATTGGTCATCGGCTGTAAA |  |  |
| *Uncharacterized protein LOC101735726* (predicted) | LOC101735726-432F | CGGCATCGAGCTTCAACAGTA | BGIBMGA013209 | XM_004922709.1 |
|  | LOC101735726-538R | TGTTCACATCACCAGGCGAA |  |  |
| *Zinc carboxypeptidase A 1-like* (predicted) | ZCarboxypeptidaseA1-323F | GGCTGTCACCAACAGTCCTCA | BGIBMGA004800 | XM_004923324.1 |
|  | ZCarboxypeptidaseA1-418R | TGTGCCACTCGAAATCCTCC |  |  |

1. Gao K, Deng XY, Qian HY, Wu P, Qin GX, et al. (2012) Novel protein of IBP from silkworm, Bombyx mori, involved in cytoplasmic polyhedrosis virus infection. J Invertebr Pathol 110: 83-91.
2. Liu J, Kolliopoulou A, Smagghe G and Swevers L (2014) Modulation of the transcriptional response of innate immune and RNAi genes upon exposure to dsRNA and LPS in silkmoth-derived Bm5 cells overexpressing BmToll9-1 receptor. J Insect Physiol.
